# Tumor Immune Evasion Induced by Dysregulation of Erythroid Progenitor Cells Development

**DOI:** 10.3390/cancers13040870

**Published:** 2021-02-19

**Authors:** Tomasz M. Grzywa, Magdalena Justyniarska, Dominika Nowis, Jakub Golab

**Affiliations:** 1 Department of Immunology, Medical University of Warsaw, 02-097 Warsaw, Poland; tomasz.grzywa@wum.edu.pl (T.M.G.); s077744@student.wum.edu.pl (M.J.); 2 Doctoral School, Medical University of Warsaw, 02-091 Warsaw, Poland; 3 Laboratory of Experimental Medicine, Medical University of Warsaw, 02-097 Warsaw, Poland

**Keywords:** immune evasion, erythroid progenitor cells, CD71+ erythroid cells, erythropoiesis, anemia, Ter-cells, ineffective erythropoiesis

## Abstract

**Simple Summary:**

Tumor immune evasion is one of the hallmarks of tumor progression that enables tumor growth despite the activity of the host immune system. It is mediated by various types of cells. Recently, immature red blood cells called erythroid progenitor cells (EPCs) were identified as regulators of the immune response in cancer. EPCs expand in cancer as a result of dysregulated erythropoiesis and potently suppress the immune response. Thus, targeting dysregulated EPC differentiation appears to be a promising therapeutic strategy.

**Abstract:**

Cancer cells harness normal cells to facilitate tumor growth and metastasis. Within this complex network of interactions, the establishment and maintenance of immune evasion mechanisms are crucial for cancer progression. The escape from the immune surveillance results from multiple independent mechanisms. Recent studies revealed that besides well-described myeloid-derived suppressor cells (MDSCs), tumor-associated macrophages (TAMs) or regulatory T-cells (Tregs), erythroid progenitor cells (EPCs) play an important role in the regulation of immune response and tumor progression. EPCs are immature erythroid cells that differentiate into oxygen-transporting red blood cells. They expand in the extramedullary sites, including the spleen, as well as infiltrate tumors. EPCs in cancer produce reactive oxygen species (ROS), transforming growth factor β (TGF-β), interleukin-10 (IL-10) and express programmed death-ligand 1 (PD-L1) and potently suppress T-cells. Thus, EPCs regulate antitumor, antiviral, and antimicrobial immunity, leading to immune suppression. Moreover, EPCs promote tumor growth by the secretion of growth factors, including artemin. The expansion of EPCs in cancer is an effect of the dysregulation of erythropoiesis, leading to the differentiation arrest and enrichment of early-stage EPCs. Therefore, anemia treatment, targeting ineffective erythropoiesis, and the promotion of EPC differentiation are promising strategies to reduce cancer-induced immunosuppression and the tumor-promoting effects of EPCs.

## 1. Introduction

Cancer immunotherapy has strongly changed the therapeutic landscape in clinical oncology, leading to significant improvements in cancer patients survival [1]. However, despite the induction of durable responses in an unprecedented percentage of cancer patients, the majority still do not respond to the treatment and eventually progress to refractory disease. There are several defined causes of immunotherapy resistance, including low tumor mutational burden [2], impaired antigen presentation by the major histocompatibility complex (MHC) proteins [3], loss of interferon-γ (IFN-γ) and tumor necrosis factor-α (TNF-α) pathway genes [4,5], as well as the development of immunosuppressive tumor microenvironment (TME) [6,7].

TME is composed of many types of cells that regulate tumor growth and progression [8]. The role of regulatory T-cells (Tregs) [9], myeloid-derived suppressor cells (MDSCs) [10], tumor-associated macrophages (TAMs) [11], tumor-associated neutrophils (TANs) [12], and cancer-associated fibroblasts (CAFs) [13] in the regulation of anti-tumor immune response has been established by many years of research (Table 1). Recent reports point to another population of cells, i.e., erythroid progenitor cells (EPCs), that regulate local and systemic immunity in cancer. These cells use similar mechanisms to immune cells and are crucial in the regulation of immune response and cancer progression.

In this review, we discuss the role of the dysregulation of erythropoiesis by cancer cells to induce immune evasion and promote cancer progression.

## 2. Regulation of Erythropoiesis

The differentiation of hematopoietic stem cells (HSCs) to erythroid cells is a stepwise process strictly regulated by multiple intrinsic and extrinsic factors (Table 2), which results in the production of over 2 × 10^11^ red blood cells (RBCs) per day and allows for the maintenance of erythroid homeostasis [44,45,46,47,48]. This complex net of interactions provides adequate production of RBCs depending on the body’s needs. Insufficient oxygen supply to the peripheral tissues resulting in hypoxia is a key trigger of increased erythropoiesis, which is regulated by the increased production of erythropoietin (EPO) in the kidney peritubular fibroblasts and liver interstitial cells and hepatocytes [49].

HSCs reside in a unique niche that is created and regulated by various cell types, growth factors, and chemokines [69]. The commitment of HSCs to erythroid lineage begins with the differentiation to a multipotent megakaryocyte–erythroid progenitor cell (MEP), followed by a bust-forming unit-erythroid (BFU-E) and colony-forming unit-erythroid (CFU-E). During terminal erythropoiesis, CFU-E differentiates into proerythroblasts, basophilic erythroblasts, polychromatic erythroblasts, and orthochromatic erythroblasts that expel their nuclei and generate reticulocytes [70]. Reticulocytes are released to the circulation, where they mature to RBCs within a few days. In healthy humans, erythroblasts represent about 20–30% of nucleated cells in the bone marrow [71,72].

The first steps of erythropoiesis are regulated by hematopoietic cytokines including stem cell factor (SCF), interleukin 3 (IL-3), insulin-like growth factor 1 (IGF-1), and granulocyte-macrophage colony-stimulating factor (GM-CSF) [73,74,75]. Further erythroid cell differentiation is regulated mainly by EPO [45,76,77]. The impairment of steady-state erythropoiesis triggers stress erythropoiesis that maintains erythroid homeostasis. Stress erythropoiesis is regulated by additional factors including hypoxia, bone morphogenetic protein 4 (BMP4), Hedgehog, glucocorticoids, and peroxisome proliferator-activated receptor α (PPAR-α) [78,79].

Cell lineage specification is regulated through defined transcriptional programs. It is well established that a zinc-finger transcription factor GATA1 is a master transcriptional regulator of differentiation toward erythroid lineage [80]. It is induced at the very early stages of erythropoiesis and is responsible for the regulation of all known erythroid genes [80]. Thus, GATA1 is necessary for erythropoiesis and its lack cannot be compensated as *Gata1* knockout mice fail to generate mature RBCs [81]. Therefore, the cleavage of GATA1 is a key mechanism of erythropoiesis regulation. GATA1 is cleaved by caspases, primarily caspase-3, which is activated in the nucleus of terminally differentiating erythroid cells to enable maturation to RBCs [80,82,83]. Nonetheless, the activation of caspases and GATA1 degradation at earlier stages of differentiation induces differentiation arrest and apoptosis. Therefore, GATA1 is protected from degradation in early-stage EPCs by EPO signaling, p19^INK4d^ cyclin-dependent kinase inhibitor, and HSP70 protein chaperone [76,82,84].

## 3. Erythroid Progenitor Cells as Immune Regulators

EPCs are predominantly erythroblasts and reticulocytes that differentiate into mature RBCs. EPCs are characterized by the expression of transferrin receptor 1 (CD71) and glycophorin A (CD235a) in humans, and CD71 and TER119 in mice [85]. For many years, EPCs were considered to be solely erythrocytes precursors, without any other significant functions in the human body. However, recent studies revealed the importance of the previously neglected role of EPCs.

Immunomodulatory functions of EPCs were described for the first time in neonates, which are characterized by a physiological enrichment of EPCs in extramedullary sites, including the spleen, liver, and peripheral blood [86]. Neonatal EPCs express arginase-2 (ARG2), _L_-arginine degrading enzyme, and secrete transforming growth factor β (TGF-β), leading to the suppression of cytokine production by myeloid cells [86] and the promotion of T-cell differentiation toward Tregs cells [87]. Despite initial hypotheses that only neonatal EPCs have significant immunoregulatory properties [86], further research expanded our knowledge and revealed that these properties are a general feature of EPCs. The regulation of immune cells by erythroid cells was described for EPCs induced by pregnancy [88], systemic inflammation [89], HIV infection [90], COVID-19 [91], and anemia [92].

EPCs in different conditions modulate immune response via various mechanisms (Table 3). Recent studies also demonstrated that EPCs that expand during cancer progression possess significant immunomodulatory properties and promote tumor growth.

## 4. The Role of Erythroid Progenitor Cells in Cancer

Cancer progression is associated with the suppression of immune response that enables tumor growth and leads to increased susceptibility to infections in patients with advanced disease [96]. It is caused by the remodeling of the immune cell landscape that impairs not only a local anti-tumor response, but also systemic antibacterial and antiviral immunity [97]. Cancer cells and tumor-associated stromal cells reprogram hematopoiesis and promote the polarization of immune cells toward suppressive phenotypes. In cancer, the spleen is a key organ of extramedullary hematopoiesis and is responsible for the production of suppressive immune cells [98]. It is well established that cancer dysregulates hematopoiesis to generate MDSCs that suppress antitumor response [10,99]. However, during tumor progression, immune cells in the murine spleen are vastly outnumbered by another type of cells, EPCs [41,62]. Moreover, substantial EPC expansion is observed in the peripheral blood and the liver of tumor-bearing mice and cancer patients. EPCs also infiltrate murine and human tumors, and their frequency in TME is much higher than that of MDSCs or Treg cells [41,42,43,62].

**Figure 1:**
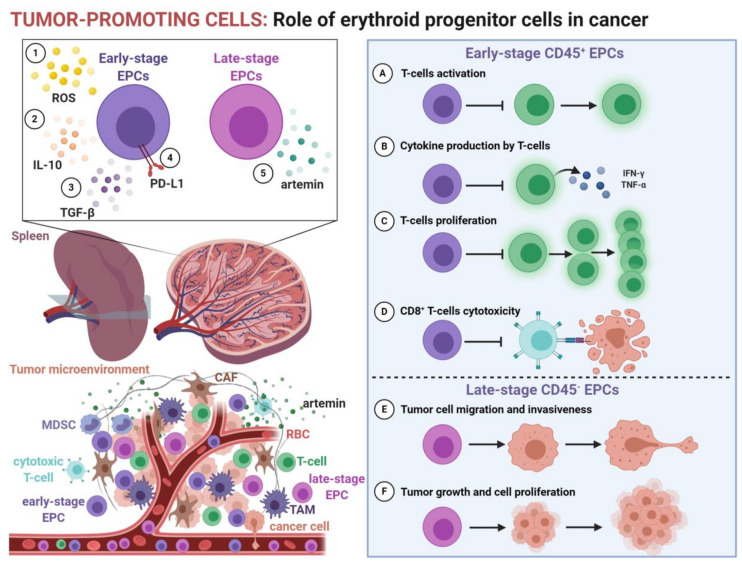
The role of erythroid progenitor cells (EPCs) in cancer. During disease progression, EPCs expand in the extramedullary sites, including the spleen. Moreover, EPCs are abundant in the peripheral blood of cancer patients and infiltrate the tumor microenvironment. Early-stage CD45^+^ EPCs use (**1**) reactive oxygen species (ROS), (**2**) interleukin-10 (IL-10), (**3**) transforming growth factor β (TGF-β), and (**4**) programmed death-ligand 1 (PD-L1) to modulate the immune response. EPCs inhibit (**A**) T-cell activation, (**B**) production of interferon γ (IFN-γ) and tumor necrosis factor α (TNF-α), (**C**) T-cell proliferation, and (**D**) cytotoxicity of CD8^+^ T-cells. More mature CD45^−^ EPCs regulate cancer progression by (**5**) secretion of a neurotropic factor, artemin. These late-stage EPCs, called Ter-cells, promote (**E**) tumor cell migration and invasiveness as well as (**F**) tumor growth and cell proliferation.

Similar to neonatal counterparts, EPCs induced by cancer were found to potently suppress immune response (Figure 1). The proliferation and cytotoxicity of CD8^+^ T-cells, as well as the proliferation of CD4^+^ T-cells and T_H_1 differentiation, are inhibited by tumor-induced murine EPCs [41]. In murine models, the depletion of EPCs with anti-CD71 antibody inhibits tumor growth [43]. Likewise, EPCs isolated from peripheral blood of cancer patients or human tumors potently suppress T-cell proliferation and the production of IFN-γ via paracrine and direct cell-to-cell contact manner [41,42].

Erythropoiesis is a continuous process by which erythroid cells change their characteristics to differentiate into specialized oxygen-transporting RBCs. The transcriptional profile [41,42,100,101,102,103,104,105,106,107] and cell proteome [106,108,109,110,111] substantially change during erythroid maturation. Growing evidence indicates that the role of EPCs in cancer changes with maturation (Table 4). During differentiation, EPCs lose expression of CD45, a pan-leukocyte marker [112]. Therefore, CD45 may be used as a marker of early-stage EPCs [41]. In tumor-bearing mice, CD45^+^ EPCs constitute over 40% of EPCs and are predominantly responsible for the immunosuppressive effects of EPCs [41]. These early-stage EPCs were found to potently suppress T-cells, in contrast to more mature CD45^−^ erythroid cells [41,62]. In mice, the suppressive capacity of CD45^+^ EPCs falls between Tregs and MDSCs [41], but in humans, CD45^+^ EPCs are even more potent immunosuppressors than both Tregs and MDSCs [42].

Transcriptional analysis revealed a close resemblance between CD45^+^ EPCs and MDSCs and enrichment in the reactive oxygen species (ROS) pathway in CD45^+^ EPCs [41]. Early stage CD45^+^ EPCs have upregulated expression of NADPH oxidase (NOX) family members [41,42], crucial ROS-generating NADPH oxidases [113]. As a result, they have increased ROS levels compared to CD45^−^ counterparts [41,42]. Although ROS are required for T-cell activation, excessive ROS levels impair T-cell immunity [114]. Thus, ROS are a well-established mechanism of T-cell suppression by MDSCs [115]. Similarly, EPCs were found to suppress T-cells in a ROS-dependent manner. Apocynin, an NADPH oxidase inhibitor, as well as N-acetylcysteine, an ROS scavenger, diminished the suppressive effects of EPCs [41,42,116]. The infiltration of EPCs to TME probably contributes to the high ROS levels triggering oxidative stress. High ROS levels in TME impair the functions of tumor-infiltrating lymphocytes and dendritic cells, while promoting the recruitment and accumulation of Tregs and MDSCs [117]. However, ROS inhibition did not restore T-cell functions completely [41]. Further studies revealed that CD45^+^ EPCs induced by cancer use multiple additional immunoregulatory mechanisms, including IL-10, TGF-β, and PD-1/PD-L1 [42,43]. Thus, the immunoregulatory functions of EPCs rely on many mechanisms identified for immunosuppressive cells in TME (Table 1).

It seems that EPCs impair both anti-tumor immunity and systemic immune response to pathogens. In mice, CD45^+^ EPCs potently inhibit the antigen-specific response of tumor-infiltrating cytotoxic T-cells [41]. The transfer of CD45^+^ EPCs into tumor-bearing mice accelerated tumor growth [41], confirming the suppression of anti-tumor response by EPCs. Likewise, CD45^+^ EPCs suppressed proliferation and cytokine production by tumor-infiltrating T-cells from cancer patients [42].

Importantly, the expansion of EPCs in cancer is most remarkable in the spleen (Table 3), which is the largest secondary lymphoid organ involved in the development of systemic immune response to blood-borne antigens [118]. Similarly to neonates that are also characterized by the expansion of EPCs in the spleen [86], adult tumor-bearing mice have increased susceptibility to viral and bacterial infections compared to healthy mice [41]. Ex vivo, EPCs suppressed antigen-specific cytotoxic T-cells [41]. In vivo, the depletion of EPCs with anti-CD71 antibody rescued the suppressed proliferation of virus-specific CD8^+^ T-cells, restoring anti-viral immunity in tumor-bearing mice while the transfer of CD45^+^ EPCs potentiated the suppression of immune response [41]. In humans, anemic cancer patients have higher EPC numbers and increased Epstein–Barr viral (EBV) load due to suppressed anti-viral immunity [41]. These latter findings suggest that as in mice, EPCs suppress a systemic immune response in cancer patients.

It was suggested that the suppressive properties of EPCs may be restricted to stress erythropoiesis-induced EPCs. However, CD45^+^ EPCs isolated from the spleen, liver as well as bone marrow of the tumor-bearing mice suppressed T-cells to a similar extent [41]. Moreover, steady-state EPCs from human bone marrow also suppress T-cells [92]. Nonetheless, there are significant differences in the expression of immunomodulatory molecules, including PD-L1, between EPCs isolated from the bone marrow, spleen, and TME [43]. This suggests that the differences in EPC properties may result from stimulation with some factors, presumably cytokines or TME components, which may enhance or diminish the immunosuppressive properties of EPCs.

### Tumor-Promoting Role of CD45^−^ EPCs

The majority of tumor-induced EPCs are CD45^−^ [41,62]. While early-stage CD45^+^ EPCs potently suppress the immune response, more mature CD45^−^ EPCs lack this capacity (Table 4). However, the transfer of CD45^−^ EPCs also promotes tumor growth and decreases the survival of tumor-bearing mice [62].

These tumor-induced splenic CD45^−^ EPCs were called Ter-Cells [62]. They are a population of late-stage EPCs as they have a high nucleus/cytoplasm ratio, scant cytoplasm, dense chromatin, and few organelles, as well as lacking the expression of the major histocompatibility complex (MHC) class I [62], a marker of mature erythroid cells [119]. In contrast to early-stage EPCs, CD45^−^ EPCs do not influence T-cell proliferation, dendritic cell activation, and cytokine secretion as well as fail to induce Tregs [62]. Moreover, CD45^−^ EPCs have very low or undetectable levels of immune-related mediators, including IL-10, TGF-β, IL-4, prostaglandin E2 (PGE-2), and ROS [41,42,62]. Therefore, CD45^−^ EPCs do not promote tumor growth by inhibiting the anti-tumor response.

Transcriptional analysis revealed marked overexpression of a neurotrophic factor artemin in CD45^−^ EPCs [62]. The physiological role of artemin involves the regulation of neuronal survival, maintenance, and differentiation [120]. Artemin also has protumorigenic activity and promotes cancer cell survival, proliferation, migration, and invasiveness [62,121,122,123]. In murine models, it promotes tumor growth and accelerates disease progression [62]. Artemin activates the glial cell-derived neurotrophic factor (GDNF) family receptor alpha-3 (GFRα3) and its co-receptor RET on cancer cells. Downstream signaling of artemin promotes the phosphorylation of extracellular signal-regulated kinase (ERK), protein kinase B (AKT), and caspase-9, promoting proliferation and invasiveness, while preventing apoptosis in tumor cells, even induced by the therapy [62]. The same effects are exerted by artemin-secreting CD45^−^ EPCs. Thus, the reduction in EPC expansion reduces the increase in the artemin concentration in the serum and decreases tumor growth [62]. Artemin-expressing CD45^−^ EPCs were also detected in the spleens of patients with hepatocellular carcinoma (HCC) and pancreatic ductal adenocarcinoma (PDAC) [62,123], which suggests their role in cancer patients.

Moreover, these differences are also manifested by their localization. While immunomodulatory early-stage EPCs accumulate in the spleen and intensively infiltrate TME, tumor-promoting late-stage EPCs are detected mainly in the spleen where they secrete artemin into circulation [41,42,62,123]. Collectively, early-stage and late-stage EPCs differ substantially regarding their gene expression profile, level of immunomodulatory mediators, and their role in promoting cancer progression (Table 5).

## 5. Expansion of Erythroid Progenitor Cells

EPCs predominantly occupy niches in the bone marrow where they differentiate into RBCs. However, EPC frequency in the steady-state bone marrow is relatively low, especially when compared to mature erythrocytes. In healthy individuals, EPCs are not detected in extramedullary sites, besides a small percentage of reticulocytes in peripheral blood [124]. However, under several conditions, EPCs substantially expand in the bone marrow as well as in extramedullary sites.

The expansion of EPCs is physiological in neonates and during pregnancy [88,93,125,126]. In neonates, EPCs accumulate in the extramedullary sites due to insufficient bone marrow erythropoiesis during the first days of life [125,126]. During pregnancy, extramedullary erythropoiesis enables the production of sufficient numbers of erythrocytes [88]. Moreover, the expansion of EPCs is also observed in anemic patients as a mechanism increasing oxygen transport [92,127]. Recent studies revealed that extramedullary erythropoiesis and EPC expansion may also be a part of the inflammatory response [128,129,130]. A recent analysis of blood transcriptome revealed that the signature of immature erythroid cells is also associated with severe respiratory syncytial virus (RSV) infection, pharmacological immunosuppression, and late-stage cancer [131].

In tumor-bearing mice, EPCs expand during tumor progression in many extramedullary organs (Table 6), predominantly the spleen, liver, and peripheral blood, as well as infiltrate tumors [41,43,62]. In humans, EPCs were detected in the spleen, TME, and peripheral blood of cancer patients [41,62,123,131]. In general, anemia severity correlates with the frequency of EPCs [41]. In some cases, the expansion of EPCs in peripheral blood is so substantial that it causes a so-called leukoerythroblastic reaction [132,133].

## 6. Cancer-Induced Dysregulation of Erythropoiesis

**Figure 2:**
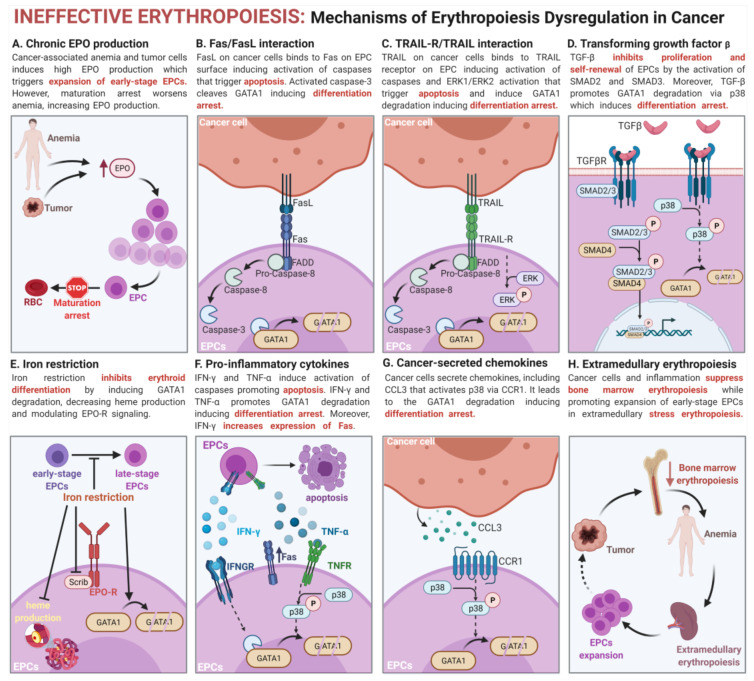
Mechanisms of erythropoiesis dysregulation in cancer. Expansion of early-stage EPCs is caused by (**A**) chronic erythropoietin (EPO) production. However, EPCs are unable to generate mature red blood cells (RBCs) due to increased apoptosis and differentiation arrest. EPCs apoptosis is triggered by (**B**) FasL/Fas and (**C**) TRAIL-TRAIL-R interaction between EPCs and cancer cells. Differentiation arrest of early-stage EPCs is caused by (**D**) transforming growth factor β (TGF-β), (**E**) iron restriction, (**F**) pro-inflammatory cytokines, and (**G**) cancer-secreted chemokines. Inhibited maturation is an effect of GATA1 degradation mediated by caspase-3 or p38 activation. (**H**) Bone marrow steady-state erythropoiesis is suppressed by inflammation and triggers stress erythropoiesis and expansion of EPCs in extramedullary sites.

The main cause of EPC expansion is the increase in EPO concentrations in response to anemia. However, the mechanisms of EPC induction by cancer are complex and rely on multiple components that dysregulate erythropoiesis (Figure 2), leading to ineffective erythropoiesis, characterized by erythroid differentiation arrest and increased apoptosis of erythroid cells, and is a feature of various diseases, including β-thalassemia [135]. Importantly, emerging evidence suggests that cancers not only induce potent EPC expansion, but also arrest their development at the earliest stages of differentiation. This leads to the suppression of immune response driven by EPCs, which are potent but physiologically transient immunosuppressors [92].

### 6.1. Dysregulation of Hematopoietic Stem and Progenitor Cells Differentiation

The dysregulation of erythropoiesis by cancer begins at the first stage of hematopoiesis. Malignant hematological cells suppress hematopoietic stem and progenitor cells (HSPCs) in the bone marrow, limiting their differentiation and inducing a quiescent state. This suppression is mediated by various mechanisms, including SCF [51], TNF-α [136], arginase [137], TGF-β [138,139,140], and stanniocalcin 1 [141]. On the other site, HSPCs are enriched in the extramedullary sites, predominantly the spleen and the circulation of cancer patients, and are myeloid-biased to generate suppressive myeloid cells [142,143,144,145,146]. Cytokines and growth factors secreted by cancer cells and TME force hematopoiesis to the generation and maintenance of immunosuppressive cells that promote tumor growth [144]. Increased numbers of HSPCs in the circulation correlate with advanced tumor stage and decreased progression-free survival in cancer patients [142,146]. In cancer, TNF-α secreted by activated T-cells increases the proliferation of HSPCs and induces emergency myelopoiesis in the bone marrow [143]. Nonetheless, despite strong myeloid polarization, HSPCs in the extramedullary sites, including the spleen, also exhibit increased capacity to differentiate into BFU-E in tumor-bearing mice compared to healthy mice [144].

### 6.2. Disruption of Hematopoietic Stem and Progenitor Cells Niche

Cancer cells also directly impair HSPCs’ maintenance and differentiation by disrupting their niche, resulting in the loss of quiescence and stemness of HSPCs [51,147]. This phenomenon is the most prominent for hematological malignancies that primarily develop in the bone marrow and outcompete native HSPC niches [148]. Nonetheless, solid tumors were also reported to disrupt the HSPCs’ niche. Melanoma cells secrete vascular endothelial growth factor (VEGF), which reduces available vascular niches in bone marrow, promoting HSC mobilization [149]. Moreover, tumor-secreted exosomes educate bone marrow cells toward a pro-metastatic phenotype [150] and promote the production of pro-inflammatory cytokines by mesenchymal stem cells to support cancer cell growth while suppressing HSPCs [151].

### 6.3. Suppression of Erythroid Differentiation of Hematopoiesis Stem Cells

The differentiation of HSCs is skewed towards myelopoiesis by cancer. Thus, the potential of erythroid differentiation of HSCs is commonly suppressed, especially in the bone marrow. EPC precursors, MEPs, are the most suppressed progenitor cells in the bone marrow of mice with hematological malignancies [57,58,136,140,152,153,154]. In plasma cell myeloma, malignant cell infiltration correlates negatively with hemoglobin concentration, but not with leukocytes or platelet counts, which suggests the selective impairment of erythropoiesis by malignant cells [155]. This suppression is partially compensated by the increased proliferation of early-stage EPCs in cancer [57]. Moreover, erythroid progenitors are activated in extramedullary sites, including the spleen [154].

### 6.4. Chronic Erythropoietin Production

EPC expansion is triggered primarily by EPO, which promotes the survival, proliferation, and differentiation of EPCs [156]. EPO induces the expansion of highly proliferative early-stage EPCs [157]. Normally, this response is rapid and EPO concentration quickly decreases after the induction of EPC expansion, which results in RBC generation [158]. However, when the erythropoietic response is insufficient to rescue anemia, EPO is produced constantly. This results in the substantial expansion of early-stage EPCs in the bone marrow as well as in the extramedullary sites. In cancer, EPO is secreted predominantly in response to tissue hypoxia resulting from anemia.

Moreover, tumors may directly trigger the upregulation of EPO production in a vascular endothelial growth factor (VEGF)-dependent mechanism. Vascular endothelial growth factor (VEGF) is a growth factor produced by malignant and stromal cells in TME to induce neovascularization, vessel remodeling [159], and to modulate antitumor immune response [160]. VEGF concentration is substantially increased in the plasma of cancer patients [161]. Importantly, VEGF stimulates EPO secretion by splenic stromal cells expressing platelet-derived growth factor receptor β (PDGFR-β) [162]. Increased VEGF concentration in plasma leads to the increased reticulocyte index in the circulation and expansion of early-stage EPCs in the bone marrow and the spleen [163].

### 6.5. Induction of Erythroid Cell Apoptosis

Another mechanism of erythropoiesis dysregulation is the direct induction of erythroid cell apoptosis by cancer cells. Apoptosis induced by death receptor Fas (CD95) and Fas ligand (FasL, CD95L, or CD178) interaction is a critical negative regulatory axis of erythropoiesis [76,82]. These negative signals can be overcome by high EPO concentrations that promote EPC expansion during erythropoietic stress response [164].

Cancer cells secrete multiple factors that induce the expression of death receptors on EPCs, increasing their susceptibility to apoptosis [59,63]. Thus, EPCs from cancer patients have significantly upregulated Fas receptor [59,63]. Moreover, ligands for cell death receptors are commonly overexpressed by malignant cells [59,63,165]. The activation of death receptors triggers the activation of caspases that cleave GATA1 transcription factors, leading to the maturation arrest or apoptosis of EPCs [166]. Maturation arrest caused by GATA1 degradation results in the accumulation of EPCs at the earliest stages of differentiation [155,166]. Moreover, GATA1 downregulation decreases the induction of anti-apoptotic proteins, including Bcl-xL and Bcl-2 [167,168]. The enhanced loss of erythroid precursors due to apoptosis leads to compensatory mechanisms and, consequently, higher percentages of early erythroblasts in the bone marrow of cancer patients [59].

Similar to FasL, malignant cells have an increased level of TNF-related apoptosis-inducing ligand (TRAIL) [59,63,169]. EPCs are characterized by the physiological expression of TRAIL receptors [170]. Their stimulation by TRAIL on cancer cells induces differentiation arrest caused by the activation of caspases and the induction of ERK1/ERK2 signaling [82,170,171].

### 6.6. Transforming Growth Factor β

TGF-β is an important cytokine that promotes tumor growth and immune evasion [172]. Its concentration is substantially increased in the TME and serum of cancer patients [62,140,172,173,174]. Overactivation of the TGF-β pathway affects not only cells in TME, but also hematopoietic cells. Indeed, in cancer patients TGF-β signaling is the most dysregulated signaling pathway in HSPCs, which leads to impaired hematopoiesis, especially erythropoiesis [140]. In erythroid cells, TGF-β potently inhibits proliferation and self-renewal, but at a low concentration it may accelerate the differentiation of late-stage EPCs by promoting mitophagy [83,175,176,177,178].

TGF-β induces the maturation arrest of early-stage EPCs by noncanonical activation of p38, which in turn triggers GATA1 degradation [57,58,140]. Moreover, TGF-β activates SMAD2 and SMAD3 via the type III TGF-β receptor, which is transiently upregulated in early-stage EPCs [178]. Indeed, EPCs in tumor-bearing mice have overactivated SMAD2 and SMAD3 [62]. Accordingly, tumor-induced expansion of EPCs is substantially reduced in Smad3-deficient mice [62]. Moreover, EPC expansion may be prevented by the treatment with neutralizing antibody against TGF-β [62]. The ability to induce EPCs is decreased in mice bearing TGF-β-deficient tumor cells; however, not completely [62]. These findings confirm that TGF-β secreted by tumor cells and also by non-malignant cells in TME is a key factor inducing EPC expansion in cancer via SMAD signaling.

Another mechanism by which TGF-β impairs erythropoiesis involves IL-33, a member of the IL-1 superfamily of cytokines. Tumor-secreted TGF-β induces the expression of IL-33 in TME [179]. Indeed, an increased concentration of IL-33 has been reported in different types of cancer and often correlates with poor prognosis [180]. Notably, IL-33 potently inhibits the differentiation of EPCs at early stages by NF-κB activation and the inhibition of signaling pathways downstream of erythropoietin receptor (EPO-R) [181].

Other members of the TGF-β superfamily, including growth differentiation factor 11 (GDF11, also known as BMP11) and GDF15, have a similar role in the regulation of erythropoiesis. GDF11 induces the differentiation arrest of early-stage EPCs by the activation of SMAD2 and SMAD3 pathways, inhibiting terminal differentiation [182,183,184]. In myelodysplastic syndrome (MDS) patients, GDF11 serum concentration is negatively correlated with late erythropoiesis [185]. Erythropoiesis is also suppressed by GDF15, which modulates iron metabolism [186].

On the other side, some members of the BMP pathway, including BMP4, are crucial regulators of stress erythropoiesis and initiate the differentiation and expansion of EPCs, enabling erythropoietic response [128,187,188].

### 6.7. Iron Restriction

Iron is an important trace element required for many biological processes, including the heme synthesis [78,189]. Thus, its metabolism is regulated by multiple proteins including iron-transporting transferrin, iron-storing ferritin, and ferroportin responsible for iron export from the cell [190]. Absolute iron deficiency is detected in over 40% of cancer patients [191]. Notably, iron restriction selectively impairs erythroid cell differentiation, but not granulocytic nor megakaryocytic progenitors [61,192,193,194]. Iron is a metabolic checkpoint that restrains the expansion of EPCs triggered by EPO in the case of insufficient iron availability. Iron restriction downregulates the crucial control element of the EPO receptor, Scribble, preventing further EPC maturation [61]. Moreover, iron control of EPC differentiation is mediated by an aconitase-associated regulatory pathway that compromises heme production and modulates EPO signaling [194]. This results in profound changes in the gene expression profile, including the downregulation of GATA1 and its target genes, leading to the impairment of EPC maturation with the differentiation arrest of early EPCs [192,193,194,195].

EPCs can obtain and concentrate iron with exceptional efficacy [196]. Nonetheless, cancer cells and nonmalignant cells in TME are also characterized by increased iron metabolism [197,198]. Cancer cells overexpress CD71 and compete with the EPCs for transferrin-bound iron [199]. Moreover, cells in TME, especially macrophages, accumulate iron, leading to its sequestration from EPCs and exaggerating iron deficiency [165].

### 6.8. Pro-Inflammatory Cytokine-Driven Erythropoiesis Impairment

Anemia of inflammation (also referred to as anemia of chronic disease) is associated with systemic inflammation, which is one of the hallmarks of cancer and is primarily caused by altered iron distribution [200,201]. Inflammation activates the inflammasome, which triggers enzymatic activation of caspases [202]. Inflammasome assembly in HSPCs leads to the GATA1 cleavage by caspase-1, which favors myelopoiesis over erythropoiesis and suppresses terminal erythropoiesis, leading to the maturation arrest of EPCs [203]. In mice expressing active *Kras^G12D^,* the activation of inflammasome leads to myeloproliferation and anemia with a compensatory expansion of EPCs in peripheral blood [204]. In this model, anemia as well as EPC expansion are reduced after pharmacological inflammasome inhibition [204].

Chronic inflammation inhibits the late-stage differentiation of EPCs, leading to the maturation arrest of the early-stage EPCs, which is mediated by various cytokines [205]. One of the critical mediators of inflammation is interferon γ (IFN-γ) [206], which also potently impairs erythropoiesis, leading to anemia [207]. Erythroid cells stimulated with IFN-γ have increased levels of pro-apoptotic caspases, induced differentiation arrest, and triggered apoptosis [208,209]. Moreover, IFN-γ upregulates the expression of Fas on EPCs, increasing their susceptibility to apoptosis in vivo [210]. Additionally, IFN-γ induces the expression of a key regulator of myeloid differentiation, PU.1, in EPCs [207]. During physiological erythropoiesis, the expression of PU.1 is downregulated due to the inhibitory effects on GATA1 functions and erythroid cell differentiation [211,212,213]. Thus, chronic IFN-γ production results in decreased erythropoietic activity in the bone marrow, but increased myelopoietic activity [207]. Moreover, IFN-γ reduces RBC life span and increases macrophage erythrophagocytosis, aggravating anemia and stimulating EPC expansion [207].

Similar suppressive effects on erythropoiesis have been described for another pro-inflammatory cytokine, TNF-α. Cancer patients are characterized by the chronic production of TNF-α, which promotes immune escape and tumor progression [214]. TNF-α induces the maturation arrest of early-stage EPCs and promotes their apoptosis [82,215,216,217,218]. This effect is mediated by the p55 TNF receptor and the activation of caspases [82,215]. TNF-α also upregulates p38 MAPK in EPCs, which phosphorylates acetylated GATA1, promoting its degradation [219,220]. Moreover, TNF-α upregulates PU.1 and GATA2 in HSPCs, which antagonize erythroid cell differentiation [221].

Likewise, the maturation arrest of EPCs at early stages and the inhibition of EPC proliferation are also triggered by other proinflammatory cytokines that are overexpressed in cancer, including IL-1 [222], IL-6 [223] or IL-12 [224].

### 6.9. Cancer-Secreted Chemokines

Erythropoiesis is also influenced by dysregulated chemokine profiles in the bone marrow plasma and serum of cancer patients. One of these chemokines is CCL3, which is upregulated in the majority of patients with hematopoietic malignancies [57,58] and a subset of patients with solid tumors [225]. CCL3 suppresses erythroid differentiation by p38 activation via CCR1, and this leads to the degradation of GATA1 [57,58,226]. On the other side, chemokines may also promote erythropoiesis by recruiting monocyte-derived macrophages to create erythroblastic islands in the extramedullary sites [227].

### 6.10. Induction of Extramedullary Stress Erythropoiesis

Suppressed bone marrow steady-state erythropoiesis is a hallmark of inflammation and is caused by the production of pro-inflammatory cytokines and iron sequestration [130,207,228,229]. Suppression steady-state erythropoiesis is often observed in patients with hematological malignancies [136,140,152,153] and solid tumors [230]. As a consequence, stress-erythropoiesis is activated in extramedullary sites to maintain erythroid homeostasis [129]. EPO secreted in response to anemia promotes the formation of erythroblastic islands in the spleen followed by the extensive proliferation of erythroid cells [228,231]. Multiple inflammatory cytokines that suppress erythropoiesis in the bone marrow simultaneously induce stress erythropoiesis. This effect was reported for IFN-γ [207], TNF-α [229], IL-1β [229,232], IL-6 [223], and G-CSF [233]. Notably, extramedullary stress erythropoiesis may be also induced by other factors, including ultraviolet B (UVB) exposure, tumor-promoting environmental stress [43], and chronic stress [234,235], which often accompany cancer.

Importantly, EPCs in extramedullary sites may still exhibit differentiation arrest that results in the enrichment of early-stage EPCs [207]. Thus, early-stage EPC fraction is increased in the spleen of tumor-bearing mice compared with acute anemic mice that also have induced stress erythropoiesis [41].

### 6.11. Chemotherapy-Induced Impairment of Erythropoiesis

The myelosuppressive effects of chemotherapy are another cause of anemia in cancer [166]. Importantly, early-stage EPCs are especially sensitive to the cytotoxic effects of chemotherapeutic agents, while late-stage EPCs are more resistant [236]. EPC apoptosis triggered by chemotherapy is induced by caspase activation and can be prevented by the SCF-mediated up-regulation of anti-apoptotic proteins Bcl-2 and Bcl-X_L_ [236]. Therefore, it was suggested that SCF may be used in the supportive therapy of chemotherapy-treated cancer patients [237]. This approach may diminish the development of anemia, which would cause the extensive expansion of EPCs after treatment.

## 7. Modulation of EPCs to Inhibit Their Tumor-Promoting Effects

The development of strategies that modulate the immune response in cancer patients is of great clinical interest. The modulation of immunosuppressive and tumor growth-promoting EPC mechanisms is a promising approach to diminish their negative role. Moreover, treating anemia to prevent EPC expansion as well as targeting ineffective erythropoiesis may causally decrease the tumor-promoting effects of EPCs.

### 7.1. Modulation of EPCs Immunosuppressive Mechanisms

#### 7.1.1. Reactive Oxygen Species

The production of ROS by EPCs is a key mechanism of immune suppression as ROS scavengers substantially rescue T-cell function suppressed by both murine and human EPCs [41,42,116]. Several antioxidant-based therapies were demonstrated to have potent antitumor effects in preclinical studies [238]. However, further studies revealed that antioxidants may accelerate tumor progression and promote metastasis [239,240]. Therefore, current studies focus on increasing rather than decreasing ROS levels in TME due to increased vulnerability to oxidative stress-induced apoptosis [241]. Indeed, ROS-generating agents or inhibitors of antioxidant systems are efficient in preclinical studies; however, they are without satisfactory results in clinical trials [242]. Thus, more research is required to determine the clinical utility of ROS-based therapies in cancer.

#### 7.1.2. IL-10

IL-10 was considered for many years as a potent anti-inflammatory cytokine. Accordingly, EPCs were found to secrete IL-10, which suppresses T-cells [42]. However, many studies in this field demonstrated that its role in cancer is more complex than initially envisioned [243,244,245,246]. Intriguingly, IL-10-based therapy, including pegylated IL-10 (Pegilodecakin), is much more efficient than therapies neutralizing IL-10 effects [246,247,248].

#### 7.1.3. PD-L1/PD-1 Axis

Targeting immune checkpoints has revolutionized clinical oncology. Monoclonal antibodies targeting PD-L1 or PD-1 reverse the inhibitory signals triggered by the PD-L1/PD-1 axis and enhance antitumor immune response [249,250,251]. PD-L1 is also expressed by murine and human tumor-induced EPCs [43]. Interestingly, the expression of PD-L1 is higher in stress erythropoiesis EPCs compared to steady-state EPCs in the bone marrow, and it reaches the highest levels in tumor-infiltrating EPCs [43]. Although an exact role of the PD-L1/PD-1 axis in the EPC-mediated suppression of immune response was not assessed, it seems that immune checkpoint inhibitors may at least partially diminish their tumor-promoting effects.

#### 7.1.4. TGF-β

The production of TGF-β in the TME is crucial to induce and maintain its immunosuppressive character [172]. TGF-β is produced by various types of cells, including EPCs [42]. The inhibition of SMAD signaling induced by TGF-β rescues T-cell proliferation and the production of IFN-γ suppressed by EPCs [42]. Therefore, modulating TGF-β signaling is a promising strategy to attenuate immune evasion induced by tumor-associated cells, including EPCs. Indeed, several anti-TGF-β-based immunotherapies were shown to be effective in preclinical studies, especially in combination with immune checkpoint inhibitors [252,253,254,255]. Therefore, targeting TGF-β signaling is a promising approach to suppress the tumor-promoting effects of EPCs.

### 7.2. Anti-Artemin Therapy

Late-stage EPCs promote tumor growth and invasiveness via the secretion of artemin [62,123]. Anti-artemin neutralizing antibody inhibits tumor growth and increases the survival of tumor-bearing mice [62]. Anti-artemin therapy is also currently tested for the treatment of cystitis-induced bladder hyperalgesia [256]. However, the clinical utility of targeting artemin or its signaling as the modulation of tumor growth-promoting effects of EPCs is unknown.

### 7.3. Treating Anemia to Prevent EPC Expansion

**Figure 3:**
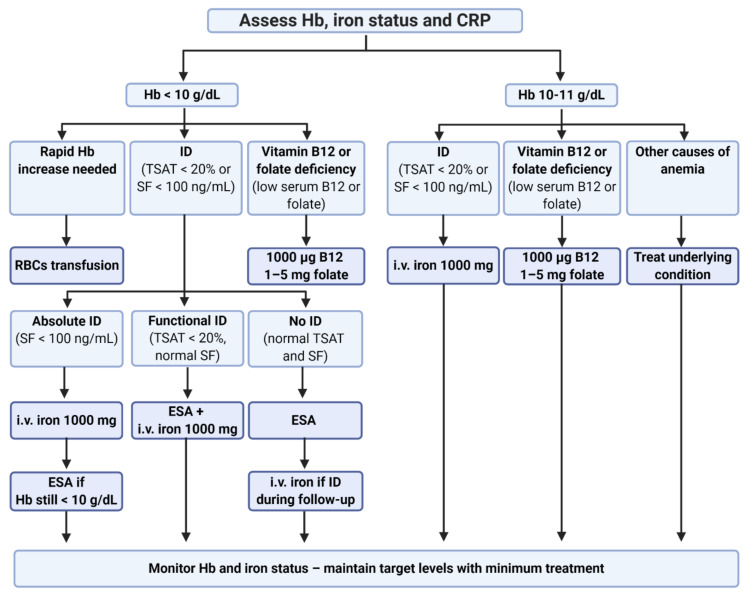
Management of anemia in cancer patients according to ESMO guidelines [258]. Additionally to TSAT and SF, the percentage of hypochromic cells (%HYPO) > 5% and reticulocytes hemoglobin content (CHr) < 28 pg can be used to determine impaired iron status. ID can be treated with oral iron only if ferritin < 30 ng/mL and CRP < 5 mg/L. CRP—C-reactive protein, ESA—erythropoiesis-stimulating agent, Hb—hemoglobin, i.v.—intravenous, ID—iron deficiency, RBCs—red blood cells, SF—serum ferritin, TSAT—transferrin saturation.

Since the modulation of EPCs’ tumor-promoting mechanisms is rather ineffective, a decrease in EPC expansion and the induction of their differentiation is a promising strategy. The correction of anemia in cancer patients is one of the strategies to prevent EPC expansion. Most anemic patients have iron deficiency (ID) [191]; therefore, the determination of iron status and treatment is recommended for cancer patients according to the guidelines [66,257,258]. Current European Society for Medical Oncology (ESMO) guidelines of anemia management in cancer patients are presented in Figure 3.

Impaired iron status can be diagnosed by total iron-binding capacity (TIBC), transferrin saturation (TSAT), or serum ferritin (SF) levels tests. TSAT enables the determination of iron status available for erythropoiesis, and its low levels together with high SF (>100 ng/mL) suggest functional iron deficiency (FID) [66]. Iron should be supplemented intravenously or orally for patients with low ferritin and without anemia of inflammation (CRP < 5 mg/L) [66].

However, anemia in cancer is not commonly caused by absolute ID, but rather results from iron sequestration (functional ID) [66,200,201]. In this group of patients, iron-replenishing strategies may not be effective. Therefore, targeting iron metabolisms with hepcidin antagonists to modulate the hepcidin-ferroportin axis is a promising treatment option [66,259]. Moreover, several novel therapies are currently under investigation for cancer-associated anemia, including ascorbic acid, androgens, BMP2 and BMP6 antagonists, as well as activin traps [66].

### 7.4. Targeting Ineffective Erythropoiesis to Decrease EPC Expansion

**Figure 4:**
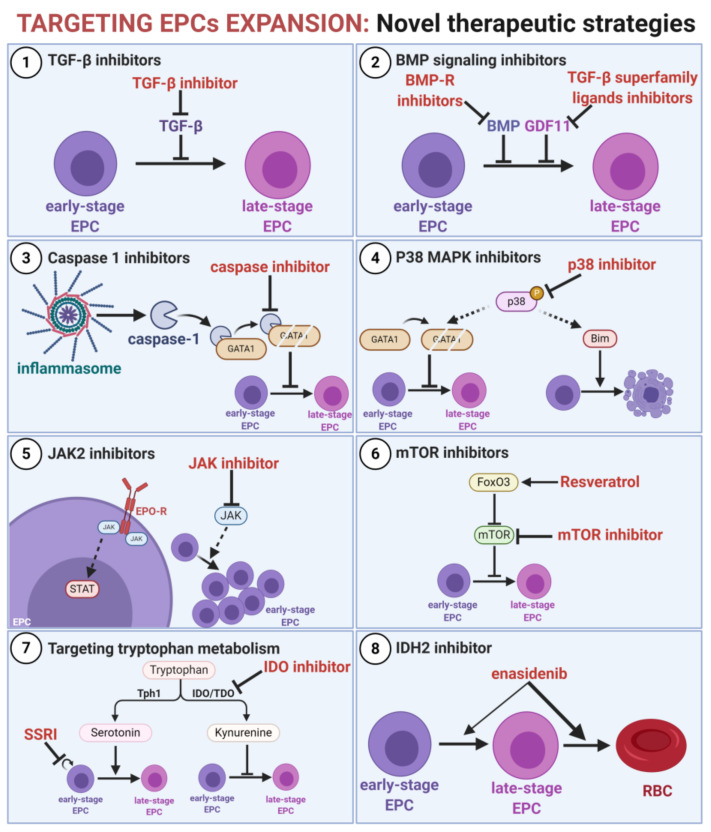
Targeting EPC expansion as a novel therapeutic strategy. Cancer-induced dysregulation of erythropoiesis resulting in the differentiation arrest of EPCs and their expansion may be diminished by different agents. Inhibitors of (**1**) transforming growth factor β (TGF-β) and (**2**) BMP signaling rescue maturation arrest. (**3**) Caspases inhibitors inhibit GATA1 cleavage triggered by the inflammasome. (**4**) P38 inhibitors promote differentiation and decrease the apoptosis of EPCs. (**5**) JAK inhibitors decrease activation of EPO-induced signaling, which decreases EPCs expansion. (**6**) mTOR inhibitors and inducers of FoxO3 promote differentiation of early-stage EPCs. (**7**) Increasing serotonin (5-HT) concentration with either selective serotonin reuptake inhibitors (SSRIs) or inhibitors of the kynurenine metabolism pathway promotes differentiation of EPCs. (**8**) Enasidenib, an inhibitor of mutated IDH2 promotes terminal differentiation of EPCs.

Modulating EPC expansion by promoting EPC differentiation is a novel therapeutic strategy to diminish the tumor-promoting effects of EPCs (Figure 4).

TGF-β is a key negative regulator of erythropoiesis, which triggers differentiation arrest and promotes the expansion of EPCs [62]. TGF-β inhibitors stimulate EPC differentiation in vitro [178,260]. In murine models, the anti-TGF-β antibody inhibits tumor growth and prevents the expansion of CD45^−^ EPCs [62]. Moreover, SMAD inhibitors rescue T-cell proliferation and IFN-γ production suppressed by EPCs [42]. Therefore, the modulation of TGF-β and SMAD2/3 signaling is a promising approach to promote erythroid cell maturation and to diminish their suppressive effects [261], despite the lack of potent antitumor effects of monotherapy in clinical trials [262].

Myelodysplastic syndromes (MDS) are characterized by the impairment of erythroid cell differentiation with maturation arrest at the early stage [263,264]. In murine MDS models, TGF-β superfamily ligand-trapping fusion protein ACE-536 promotes erythroid maturation by binding GDF11, inhibiting SMAD2/3 signaling, and promoting late-stage erythropoiesis, reducing rescue anemia [182]. Similar effects are exerted by the compound ACE-011, which promotes terminal erythropoiesis and prevents EPC expansion in β-thalassemia [184]. In clinical trials, luspatercept (ACE-536) and sotatercept (ACE-011) reduced the severity of anemia in patients with MDS and β-thalassemia [265,266,267]. Clinical trials of sotatercept in cancer patients showed that it may also be effective for the treatment of chemotherapy-induced anemia [268].

Moreover, it was suggested that targeting BMP signaling may be beneficial for anemia of inflammation [269]. The inhibition of BMP downregulates IL-6 signaling and decreases hepcidin levels, resulting in the restoration of erythropoiesis suppressed by inflammation [270,271].

Caspase-1 activation by inflammasome is one of the mechanisms skewing the differentiation of HSPCs toward myeloid cells [203]. Thus, the inhibition of caspase-1 results in the upregulation of GATA1 and the rescue of inflammation-induced anemia [203]. Moreover, caspase inhibitors trigger the differentiation of EPCs after the induction of maturation arrest by FasL or TNF-α [82]. Several caspase-1 inhibitors are available, including a potent and selective inhibitor, VX-765 [272]. Therefore, it is of great interest to evaluate the effects of caspase inhibitors on EPC expansion and differentiation in cancer.

P38 MAPK is an important pathway regulating erythropoiesis. P38 is activated by multiple inflammatory signals and restrains EPC differentiation by GATA1 degradation [220] and by suppressing the silencing of Bim, which triggers apoptosis [273]. Moreover, p38 functions as an oncogenic kinase with a complex role in cancer [274]. Therefore, p38 inhibition may simultaneously have an antitumor effect and promote EPC maturation. Notably, p38 inhibition enhances EPC maturation in an EPO-independent manner [273], but also decreases the production of endogenous EPO under stress conditions [275], suggesting that p38 inhibitor therapy should probably be combined with ESAs.

In erythroid cells, EPO triggers EPO-R and association with cytoplasmic Janus kinase 2 (JAK2), a crucial signal transducer [276]. The overactivation of JAK2, most commonly caused by V617F mutation, is associated with myeloproliferative neoplasms, including polycythemia vera [277]. Nonetheless, increased JAK2 activity may also be caused by high levels of EPO caused by anemia and chronic hypoxia, a state observed in β-thalassemia and cancer. In β-thalassemia, JAK2 inhibitors decrease ineffective erythropoiesis, prevent the expansion of EPCs, and reduce splenomegaly [278]. JAK2 inhibitors, including ruxolitinib and fedratinib, are approved for the treatment of patients with MPNs [279]. Moreover, targeting JAKs is a promising therapeutic strategy for the treatment of different types of cancer [280]. Whether JAKs inhibitors may decrease the expansion of EPCs in cancer patients remains unknown.

The mechanistic target of rapamycin (mTOR) is a central protein kinase orchestrating cell growth, metabolism, and immune response. Thus, mTOR is widely tested in clinical trials as a target for cancer therapy [281]. Importantly, mTOR inhibition rescues EPC differentiation under ineffective erythropoiesis by inducing the cell cycle exit of early-stage EPCs [282]. Moreover, mTOR inhibition in EPCs may be triggered by Forkhead-box-class-O3 (FoxO3) [282]. The activation of FoxO3 by resveratrol induces early erythroid maturation and decreases their proliferation, resulting in a reduction in ineffective erythropoiesis [283].

Erythropoiesis is also regulated by serotonin (5-HT) [284,285]. Dysregulated tryptophan metabolism with an enhanced kynurenine pathway is common in cancer, and is increasingly being recognized as a viable metabolic pathway regulating immune response [286]. A skew towards the kynurenine pathway leads to decreased serotonin (5-HT) concentrations, resulting in the impaired differentiation and decreased survival of EPCs [284,285]. The upregulation of 5-HT triggered by EPO is crucial to protect EPCs from apoptosis at the CFU-E-to-proerythroblast transition checkpoint [284]. Pharmacological increase in 5-HT with fluoxetine, a selective serotonin reuptake inhibitor (SSRI), rescues anemia [284]. Therefore, targeting the 5-HT axis in EPCs with either SSRI or kynurenine pathway inhibitors may diminish the tumor-promoting role of immature erythroid cells.

Enasidenib is an Food and Drug Administration (FDA)-approved, first-in-class preferential inhibitor of mutated isocitrate dehydrogenase 2 (IDH2) that promotes the differentiation of acute myeloid leukemia blasts [287]. Interestingly, enasidenib was found to act independently of IDH2 on EPCs. Enasidenib potently promotes erythroid differentiation through the modulation of protoporphyrin IX (PPIX) accumulation and hemoglobin production in late-stage EPCs [288]. As a result, increased hemoglobin concentration and RBC transfusion independence were reported for enasidenib-treated patients [287,289].

Some studies suggested that natural compounds may decrease EPC expansion. Dangguibuxue decoction (DGBX), a traditional Chinese medicine, abolishes EPC accumulation, promotes their differentiation, and rescues anemia, leading to the activation of anti-tumor immune response and a decrease in tumor growth [290]. Moreover, a recent study revealed that vitamin C has a critical role in the regulation of late-stage erythropoiesis and is able to rescue ineffective erythropoiesis [291].

### 7.5. Splenectomy

In cancer, the spleen becomes a central organ of extramedullary hematopoiesis, responsible for the generation of suppressive cells including EPCs and myeloid cells [292]. Therefore, it was suggested that splenectomy could be beneficial for cancer patients. In preclinical models, splenectomy inhibits tumor growth and prolongs the survival of tumor-bearing mice [62]. It also abolishes the induction of EPC expansion in extramedullary sites [62]. Similarly, splenectomy leads to the depletion of MDSCs, enhancing the activation of antitumor immunity [293]. Intriguingly, splenectomy before tumor inoculation or during tumor progression attenuates the decrease in RBC count and hemoglobin concentration, alleviating anemia [62].

However, clinical data are much less promising. Randomized trials showed that splenectomy in cancer patients not only has no advantages, but is also associated with increased perioperative morbidity [294,295,296]. Therefore, more preclinical and clinical studies are required to evaluate the effects of splenectomy in cancer.

## 8. Clinical Consequences of Tumor-Induced Anemia and EPC Expansion

Anemia is very common in cancer patients. Its prevalence differs from 30–90% depending on the type of cancer as well as the diagnostic criteria. It substantially decreases the quality of life of cancer patients [296,297]. Moreover, anemia is associated with shorter survival for patients with different types of cancer and a 65% overall increase in the risk of mortality compared to non-anemic cancer patients [191,298,299]. Importantly, severe anemia is associated with hypoxia in the TME of both primary and metastatic tumors [132], which is a known driver of aggressive tumor phenotype [300].

Clinical outcomes of EPC expansion are still unclear. In cancer patients, EPC expansion is the most prominent in individuals with moderate or severe anemia [41]. Moreover, the expansion of CD45^+^ EPCs correlates with a higher EBV load and suppressed T-cell response against the major antigenic EBV proteins, LMP2 and EBNA1 [41]. A recent study also demonstrated that CD45^−^ EPCs may have clinical significance. In PDAC patients, the counts of CD45^−^ EPCs in the spleen are increased compared with noncancerous pancreatic tumors or benign pancreatic masses [123]. High CD45^−^ EPC counts predicted poor prognosis and were associated with larger tumor size and lymph node metastases [123]. Moreover, increased serum artemin concentrations, as well as increased expression of its receptors, correlate with poor prognosis in cancer patients [62,123]. Collectively, these observations demonstrate that in cancer patients, early-stage CD45^+^ EPCs may suppress the immune response, and late-stage CD45^−^ EPCs may promote tumor growth by the secretion of artemin. Nonetheless, more research is required to accurately dissect the clinical role of EPCs in cancer patients.

## 9. Conclusions

In recent years, we have expanded our knowledge regarding the mechanisms of tumor evasion induced by dysregulation in hematopoiesis. The initial assumption that cancer only significantly regulates myelopoiesis turned out to be an oversimplification. Emerging evidence demonstrates that harnessing erythroid lineage cells together with megakaryocytes and platelets [301,302] is critical for cancer progression and immune evasion. EPCs promote tumor growth by either suppressing anti-tumor immune response or secreting growth factors, depending on the developmental stage. EPCs share many similarities with well-described suppressive cells of the immune system and use the same mechanisms to regulate the immune response.

However, several issues remain unclear and need to be investigated. First, despite differences in the expression of the immunomodulatory molecules [43], factors regulating the immunosuppressive properties of EPCs are unknown. Presumably, tumor-secreted cytokines and TME may potentiate the tumor-promoting role of EPCs. Moreover, it remains elusive whether and, if so, what factors promote the recruitment of EPCs to TME. Additionally, it is not known what are the interactions between EPCs and other cells in TME, including MDSCs, TAMs or NK cells, and comprehensive studies on the role of EPCs in TME are currently limited by technological advances. Recently, transcriptional profiling at a single-cell level has revolutionized our understanding of the complexity of cell interactions in TME, and indicates further research directions [303]. However, most of the protocols involve extensive hypotonic lysis of red blood cells [304], which drastically reduces the number of EPCs [305], excluding them from analyses of TME networks. Therefore, there is a great need to define the whole landscape of TME that includes EPCs.

Future research should focus on the comprehensive characterization of immunomodulatory mechanisms of EPCs and their regulation to better understand their function in tumor immune evasion and to enable targeting them in immunotherapy. It remains unknown whether EPCs in cancer may induce Treg differentiation, similar to their neonatal counterparts [87]. Similarly, regardless of a well-established role of EPCs in neonates [86,94], the regulation of myeloid cell response by EPCs in cancer is unknown. Moreover, erythroid cells were reported to produce IL-1β, IL-2, IL-4, IL-6, IFN-γ, and TNF-α [306]; however, their role in immune regulation by EPCs remains unknown.

It needs to be determined whether and how EPCs contribute to the clinical outcome of cancer patients undergoing various types of treatment, including immunotherapy. It was reported that EPCs may contribute to the drug resistance of cancer cells [116]. Therefore, targeting EPCs or their effector mechanisms in combination with other therapies may improve therapeutic effectiveness. Finally, the development and clinical testing of agents that could rescue erythroid maturation under cancer-induced EPC differentiation arrest are of great interest. Until then, anemia treatment is the best strategy to reduce EPC expansion and differentiation arrest, as well as to minimalize the tumor-promoting role of EPCs and to improve the survival and quality of life of cancer patients.

## Figures and Tables

**Table 1:** Immunomodulatory cells in cancer and their mechanisms of immune regulation.

Cells	Mechanisms	Effects	Ref
Regulatory T-cells (Tregs)	IL-10	T-cell suppression	[14]
	IL-2 consumption	T-cell suppression	[15]
	COX-2 and PGE2	T-cell suppression	[16]
	Adenosine	T-cell suppression	[17]
Myeloid-derived suppressor cells (MDSCs)	ARG1	T-cell suppression	[18]
	IDO	T-cell suppression Tregs induction NK cell suppression	[19,20]
	PD-L1/PD-1	T-cell suppression	[21]
	IL-10	Tregs induction	[22]
	TGF-β	Tregs induction	[22]
	CD40/CD40L	Tregs activation	[23]
	Depletion of cystine and cysteine	T-cell suppression	[24]
	ROS	T-cell suppression	[25]
	Free radical peroxynitrite	Resistance to cytotoxic T-cells	[26]
Tumor associated macrophages (TAMs)	PD-L1/PD-1	Decreased phagocytosis	[27]
	ARG1	T-cell suppression	[28]
	IL-10	T-cell suppression	[29]
	IL-1β	MDSC infiltration Induction of the protumor phenotype	[30,31]
	IL-12	Induction of T-cell response	[32]
	TNF-α	Induction of anti-tumor response	[33]
Tumor associated neutrophils (TANs)	ARG1	T-cell suppression	[18,28]
	NOS	T-cell suppression T-cell apoptosis	[34,35]
	PD-L1/PD-1	T-cell suppression	[36]
Cancer associated fibroblasts (CAFs)	PD-L1/PD-1	T-cell suppression	[37]
	FasL, PD-L2	T-cell suppression	[38]
	IL-6	Induction of PD-L1^+^ TANs	[39]
	Chemokines	MDSC infiltration	[40]
Erythroid progenitor cells (EPCs)	ROS	T-cell suppression	[41,42]
	IL-10	T-cell suppression	[42]
	PD-L1/PD-1	T-cell suppression	[43]
	TGF-β	T-cell suppression	[42]

ARG1—arginase 1, COX-2—cyclooxygenase-2, FasL—Fas ligand (CD95L, CD178), IDO—Indoleamine-pyrrole 2,3-dioxygenase, IL—interleukin, NK—natural killer, NOS—nitric oxide synthase, PD-1—programmed cell death 1, PD-L1—programmed death-ligand 1, PGE2—Prostaglandin E_2_, ROS—reactive oxygen species, TGF-β—transforming growth factor β, TNF-α—tumor necrosis factor α.

**Table 2:** Regulation of erythropoiesis.

Factor	Role in Erythropoiesis	Dysregulation in Cancer	References
SCF	Growth factors regulating early stages of erythropoiesis	Production in TME Increased serum concentration	[50,51]
G-CSF			[52]
IL-3			[53]
EPO	Growth factors regulating late stages of erythropoiesis	Increased serum concentration	[54]
GDF11		Production in TME	[55]
Activin A		Production in TME	[56]
GATA1	Crucial TFs regulating erythropoiesis	Decreased expression in EPCs in cancer	[57,58,59]
STAT5		Increased in EPCs in MPNs Decreased in EPCs in iron deficiency	[60,61]
MCL-1	Survival factors for erythroid cells		
BCL-xL			
HSP70			
TGF-β	Negative regulators of erythropoiesis	Production in TME Increased concentration	[62]
SMAD signaling		Increased level in EPCs in cancer	[62]
FasL		High expression on cancer cells	[59,63]
Fas		Increased level in EPCs in cancer	[59,63]
Vitamin B12	Essential vitamins, trace elements, and iron-metabolism proteins	Decreased in a subset of patients	[64]
Folic Acid		Decreased in a subset of patients	[64]
Copper		Increased concentration	[65]
Iron		Decreased in a subset of patients	[66]
Ferritin		Decreased or increased	[66]
Transferrin		Decreased in a subset of patients	[66]
Ferroportin		Decreased expression	[67]
Hepcidin		Increased concentration	[68]

MPN—myeloproliferative neoplasm, TF—transcription factor, TME—tumor microenvironment.

**Table 3:** Mechanisms of immunomodulatory functions of EPCs.

Source	Mechanism	Effect	Mouse	Humans	Ref.
Neonates	ARG2	↓cytokine production bymyeloid cells	+	+	[86,93]
	TGF-β	↑Tregs differentiation	+	+	[87]
	ROS	↓cytokine production bymyeloid cells ↓cytokine production by T-cells	-	+	[94]
	PD-1/PD-L1	↓cytokine production by T-cells	+	+	[88]
Pregnancy	ARG2	↓cytokine production bymyeloid cells	+	+	[24,93]
	TGF-β	↑Tregs differentiation	n.d.	+	[93]
	ROS	↓cytokine production bymyeloid cells ↓cytokine production by T-cells	n.d.	+	[93]
	PD-1/PD-L1	↓cytokine production by T-cells	+	+	[88]
Inflammatory diseases	EPCs phagocytosis	↓cytokine production byred pulp macrophages	+	n.d.	[89]
HIV-infected patients	ROS	↑HIV replication in T-cells ↑HIV trans-infection	n.d.	+	[90]
COVID-19 patients	ARG1	↓cytokine production by T-cells ↓T-cell proliferation	n.d.	+	[95]
	ARG2	↓cytokine production by T-cells ↓T-cell proliferation	n.d.	+	[95]
	ROS	↓cytokine production by T-cells ↓T-cell proliferation	n.d.	+	[95]
Anemia	ARG1	↓cytokine production by T-cells ↓T-cell proliferation	-	+	[92]
	ARG2	↓cytokine production by T-cells ↓T-cell proliferation	+	+	[92]
	ROS	↓cytokine production by T-cells ↓T-cell proliferation	+	+	[92]
Cancer	TGF-β	↓T-cells proliferation↓cytokine production by T-cells	n.d.	+	[42]
	ROS	↓T-cell proliferation↓cytokine production by T-cells	+	+	[41,42]
	PD-L1/PD-1	↓cytokine production by T-cells	+	+	[43]
	IL-10	↓T-cell proliferation↓cytokine production by T-cells	n.d.	+	[42]

↑—promoted, ↓—suppressed, n.d.—no data, - — no role, +—reported mechanism.

**Table 4:** Differences in immune-related mediators between early-stage and late-stage EPCs [41,42,43,62].

Feature	Early-Stage EPCs (CD45^+^)	Late-Stage EPCs (CD45^-^)
ROS level	↑	↓
IL-10	↑	↓
TGF-β	↑	↓
ROS pathway	↑	↓
IL-10 pathway	↑	↓
TGF-β pathway	↑	↓
PD-1/PD-L1	n.d.	n.d.
ARG2	n.d.	n.d.

↑—increased, ↓—decreased, n.d.—no data.

**Table 5:** Different role of early-stage and late-stage EPCs in cancer [41,42,43,62,116,123].

Process	Early-Stage EPCs (CD45^+^)	Late-Stage EPCs (CD45^-^)
T-cell proliferation	↓ suppressed	↔ no effect
Production of IFN-γ by T-cells	↓ suppressed	↔ no effect
Production of TNF-α by T-cells	↓ suppressed	↔ no effect
CD8^+^ T-cells cytotoxicity	↓ suppressed	↔ no effect
Dendritic cells activation	n.d.	↔ no effect
Production of IL-6 and IL-12 by dendritic cells	n.d.	↔ no effect
Tregs induction	n.d.	↔ no effect
Anti-tumor immune response	↓ suppressed	↔ no effect
Activation of signaling pathways in tumor cells	n.d.	↑ promoted
Regulation of cancer cell metabolism	↑ promoted	n.d.
Tumor cells proliferation	n.d.	↑ promoted
Tumor cells invasiveness	n.d.	↑ promoted
Tumor growth	↑ promoted	↑ promoted

↑—promoted, ↓—suppressed, ↔—no effect, n.d.—no data.

**Table 6:** The frequency of EPCs in tumor-bearing mice and cancer patients.

Organ	Mice		Humans		Ref.
	Healthy	Tumor-Bearing	Healthy	Cancer Patient	
Peripheral blood	5%	60%	0.13%	2–4.25%	[41,42]
Spleen	5%	20–50%	0.02%	0.15%	[41,43,62,123]
Bone marrow	15–20%	55%	14%	n.d.	[41,43,134]
Liver	10%	2–30%	2.5%	10%	[41,42,62]
Lymph node	1%	1%	n.d	n.d	[41,62]
Tumor	-	2–10%	-	10%	[41,42,62]

n.d.—no data, - — not applicable.

## References

[1] Waldman A.D., Fritz J.M., Lenardo M.J. (2020). A guide to cancer immunotherapy: From T cell basic science to clinical practice. Nat. Rev. Immunol..

[2] Samstein R.M., Lee C.H., Shoushtari A.N., Hellmann M.D., Shen R., Janjigian Y.Y., Barron D.A., Zehir A., Jordan E.J., Omuro A. (2019). Tumor mutational load predicts survival after immunotherapy across multiple cancer types. Nat. Genet..

[3] Rodig S.J., Gusenleitner D., Jackson D.G., Gjini E., Giobbie-Hurder A., Jin C., Chang H., Lovitch S.B., Horak C., Weber J.S. (2018). MHC proteins confer differential sensitivity to CTLA-4 and PD-1 blockade in untreated metastatic melanoma. Sci. Transl. Med..

[4] Gao J., Shi L.Z., Zhao H., Chen J., Xiong L., He Q., Chen T., Roszik J., Bernatchez C., Woodman S.E. (2016). Loss of IFN-γ Pathway Genes in Tumor Cells as a Mechanism of Resistance to Anti-CTLA-4 Therapy. Cell.

[5] Kearney C.J., Vervoort S.J., Hogg S.J., Ramsbottom K.M., Freeman A.J., Lalaoui N., Pijpers L., Michie J., Brown K.K., Knight D.A. (2018). Tumor immune evasion arises through loss of TNF sensitivity. Sci. Immunol..

[6] Murciano-Goroff Y.R., Warner A.B., Wolchok J.D. (2020). The future of cancer immunotherapy: Microenvironment-targeting combinations. Cell Res..

[7] Van Elsas M.J., van Hall T., van der Burg S.H. (2020). Future Challenges in Cancer Resistance to Immunotherapy. Cancers.

[8] Hinshaw D.C., Shevde L.A. (2019). The Tumor Microenvironment Innately Modulates Cancer Progression. Cancer Res..

[9] Togashi Y., Shitara K., Nishikawa H. (2019). Regulatory T cells in cancer immunosuppression—Implications for anticancer therapy. Nat. Rev. Clin. Oncol..

[10] Gabrilovich D.I., Ostrand-Rosenberg S., Bronte V. (2012). Coordinated regulation of myeloid cells by tumours. Nat. Rev. Immunol..

[11] Mantovani A., Marchesi F., Malesci A., Laghi L., Allavena P. (2017). Tumour-associated macrophages as treatment targets in oncology. Nat. Rev. Clin. Oncol..

[12] Shaul M.E., Fridlender Z.G. (2019). Tumour-associated neutrophils in patients with cancer. Nat. Rev. Clin. Oncol..

[13] Sahai E., Astsaturov I., Cukierman E., DeNardo D.G., Egeblad M., Evans R.M., Fearon D., Greten F.R., Hingorani S.R., Hunter T. (2020). A framework for advancing our understanding of cancer-associated fibroblasts. Nat. Rev. Cancer.

[14] Stewart C.A., Metheny H., Iida N., Smith L., Hanson M., Steinhagen F., Leighty R.M., Roers A., Karp C.L., Müller W. (2013). Interferon-dependent IL-10 production by Tregs limits tumor Th17 inflammation. J. Clin. Invest..

[15] Busse D., de la Rosa M., Hobiger K., Thurley K., Flossdorf M., Scheffold A., Höfer T. (2010). Competing feedback loops shape IL-2 signaling between helper and regulatory T lymphocytes in cellular microenvironments. Proc. Natl. Acad. Sci. USA.

[16] Yuan X.L., Chen L., Li M.X., Dong P., Xue J., Wang J., Zhang T.T., Wang X.A., Zhang F.M., Ge H.L. (2010). Elevated expression of Foxp3 in tumor-infiltrating Treg cells suppresses T-cell proliferation and contributes to gastric cancer progression in a COX-2-dependent manner. Clin. Immunol..

[17] Sundström P., Stenstad H., Langenes V., Ahlmanner F., Theander L., Ndah T.G., Fredin K., Börjesson L., Gustavsson B., Bastid J. (2016). Regulatory T Cells from Colon Cancer Patients Inhibit Effector T-cell Migration through an Adenosine-Dependent Mechanism. Cancer Immunol. Res..

[18] Grzywa T.M., Sosnowska A., Matryba P., Rydzynska Z., Jasinski M., Nowis D., Golab J. (2020). Myeloid Cell-Derived Arginase in Cancer Immune Response. Front. Immunol..

[19] Yu J., Du W., Yan F., Wang Y., Li H., Cao S., Yu W., Shen C., Liu J., Ren X. (2013). Myeloid-derived suppressor cells suppress antitumor immune responses through IDO expression and correlate with lymph node metastasis in patients with breast cancer. J. Immunol..

[20] Della Chiesa M., Carlomagno S., Frumento G., Balsamo M., Cantoni C., Conte R., Moretta L., Moretta A., Vitale M. (2006). The tryptophan catabolite L-kynurenine inhibits the surface expression of NKp46- and NKG2D-activating receptors and regulates NK-cell function. Blood.

[21] Noman M.Z., Desantis G., Janji B., Hasmim M., Karray S., Dessen P., Bronte V., Chouaib S. (2014). PD-L1 is a novel direct target of HIF-1α, and its blockade under hypoxia enhanced MDSC-mediated T cell activation. J. Exp. Med..

[22] Huang B., Pan P.Y., Li Q., Sato A.I., Levy D.E., Bromberg J., Divino C.M., Chen S.H. (2006). Gr-1^+^CD115^+^ immature myeloid suppressor cells mediate the development of tumor-induced T regulatory cells and T-cell anergy in tumor-bearing host. Cancer Res..

[23] Pan P.Y., Ma G., Weber K.J., Ozao-Choy J., Wang G., Yin B., Divino C.M., Chen S.H. (2010). Immune stimulatory receptor CD40 is required for T-cell suppression and T regulatory cell activation mediated by myeloid-derived suppressor cells in cancer. Cancer Res..

[24] Srivastava M.K., Sinha P., Clements V.K., Rodriguez P., Ostrand-Rosenberg S. (2010). Myeloid-derived suppressor cells inhibit T-cell activation by depleting cystine and cysteine. Cancer Res..

[25] Kusmartsev S., Nefedova Y., Yoder D., Gabrilovich D.I. (2004). Antigen-specific inhibition of CD8^+^ T cell response by immature myeloid cells in cancer is mediated by reactive oxygen species. J. Immunol..

[26] Lu T., Ramakrishnan R., Altiok S., Youn J.-I., Cheng P., Celis E., Pisarev V., Sherman S., Sporn M.B., Gabrilovich D. (2011). Tumor-infiltrating myeloid cells induce tumor cell resistance to cytotoxic T cells in mice. J. Clin. Invest..

[27] Gordon S.R., Maute R.L., Dulken B.W., Hutter G., George B.M., McCracken M.N., Gupta R., Tsai J.M., Sinha R., Corey D. (2017). PD-1 expression by tumour-associated macrophages inhibits phagocytosis and tumour immunity. Nature.

[28] Rodriguez P.C., Quiceno D.G., Zabaleta J., Ortiz B., Zea A.H., Piazuelo M.B., Delgado A., Correa P., Brayer J., Sotomayor E.M. (2004). Arginase I production in the tumor microenvironment by mature myeloid cells inhibits T-cell receptor expression and antigen-specific T-cell responses. Cancer Res..

[29] Ruffell B., Chang-Strachan D., Chan V., Rosenbusch A., Ho C.M.T., Pryer N., Daniel D., Hwang E.S., Rugo H.S., Coussens L.M. (2014). Macrophage IL-10 Blocks CD8^+^ T Cell-Dependent Responses to Chemotherapy by Suppressing IL-12 Expression in Intratumoral Dendritic Cells. Cancer Cell.

[30] Kaplanov I., Carmi Y., Kornetsky R., Shemesh A., Shurin G.V., Shurin M.R., Dinarello C.A., Voronov E., Apte R.N. (2019). Blocking IL-1β reverses the immunosuppression in mouse breast cancer and synergizes with anti–PD-1 for tumor abrogation. Proc. Natl. Acad. Sci. USA.

[31] Chittezhath M., Dhillon M.K., Lim J.Y., Laoui D., Shalova I.N., Teo Y.L., Chen J., Kamaraj R., Raman L., Lum J. (2014). Molecular Profiling Reveals a Tumor-Promoting Phenotype of Monocytes and Macrophages in Human Cancer Progression. Immunity.

[32] Vom Berg J., Vrohlings M., Haller S., Haimovici A., Kulig P., Sledzinska A., Weller M., Becher B. (2013). Intratumoral IL-12 combined with CTLA-4 blockade elicits T cell-mediated glioma rejection. J. Exp. Med..

[33] Kratochvill F., Neale G., Haverkamp J.M., Van de Velde L.-A., Smith A.M., Kawauchi D., McEvoy J., Roussel M.F., Dyer M.A., Qualls J.E. (2015). TNF Counterbalances the Emergence of M2 Tumor Macrophages. Cell Rep..

[34] Coffelt S.B., Kersten K., Doornebal C.W., Weiden J., Vrijland K., Hau C.S., Verstegen N.J.M., Ciampricotti M., Hawinkels L., Jonkers J. (2015). IL-17-producing γδ T cells and neutrophils conspire to promote breast cancer metastasis. Nature.

[35] Michaeli J., Shaul M.E., Mishalian I., Hovav A.H., Levy L., Zolotriov L., Granot Z., Fridlender Z.G. (2017). Tumor-associated neutrophils induce apoptosis of non-activated CD8 T-cells in a TNFα and NO-dependent mechanism, promoting a tumor-supportive environment. Oncoimmunology.

[36] Wang T.T., Zhao Y.L., Peng L.S., Chen N., Chen W., Lv Y.P., Mao F.Y., Zhang J.Y., Cheng P., Teng Y.S. (2017). Tumour-activated neutrophils in gastric cancer foster immune suppression and disease progression through GM-CSF-PD-L1 pathway. Gut.

[37] Nazareth M.R., Broderick L., Simpson-Abelson M.R., Kelleher R.J., Yokota S.J., Bankert R.B. (2007). Characterization of human lung tumor-associated fibroblasts and their ability to modulate the activation of tumor-associated T cells. J. Immunol..

[38] Lakins M.A., Ghorani E., Munir H., Martins C.P., Shields J.D. (2018). Cancer-associated fibroblasts induce antigen-specific deletion of CD8^+^ T Cells to protect tumour cells. Nat. Commun..

[39] Cheng Y., Li H., Deng Y., Tai Y., Zeng K., Zhang Y., Liu W., Zhang Q., Yang Y. (2018). Cancer-associated fibroblasts induce PDL1+ neutrophils through the IL6-STAT3 pathway that foster immune suppression in hepatocellular carcinoma. Cell Death Dis..

[40] Kumar V., Donthireddy L., Marvel D., Condamine T., Wang F., Lavilla-Alonso S., Hashimoto A., Vonteddu P., Behera R., Goins M.A. (2017). Cancer-Associated Fibroblasts Neutralize the Anti-tumor Effect of CSF1 Receptor Blockade by Inducing PMN-MDSC Infiltration of Tumors. Cancer Cell.

[41] Zhao L., He R., Long H., Guo B., Jia Q., Qin D., Liu S.-Q., Wang Z., Xiang T., Zhang J. (2018). Late-stage tumors induce anemia and immunosuppressive extramedullary erythroid progenitor cells. Nat. Med..

[42] Chen J., Qiao Y.-D., Li X., Xu J.-L., Ye Q.-J., Jiang N., Zhang H., Wu X.-Y. (2020). Intratumoral CD45^+^ CD71^+^ erythroid cells induce immune tolerance and predict tumor recurrence in hepatocellular carcinoma. Cancer Lett..

[43] Sano Y., Yoshida T., Choo M.-K., Jiménez-Andrade Y., Hill K.R., Georgopoulos K., Park J.M. (2020). Multiorgan Signaling Mobilizes Tumor-Associated Erythroid Cells Expressing Immune Checkpoint Molecules. Mol. Cancer Res..

[44] Hurwitz S.N., Jung S.K., Kurre P. (2020). Hematopoietic stem and progenitor cell signaling in the niche. Leukemia.

[45] Peter V., Guntram B., Igor T., Iris Z.U., Ulrich G., Reinhard S., Karl S., Wolfgang F., Peter B., Michael P. (2018). Normal and pathological erythropoiesis in adults: From gene regulation to targeted treatment concepts. Haematologica.

[46] Hattangadi S.M., Wong P., Zhang L., Flygare J., Lodish H.F. (2011). From stem cell to red cell: Regulation of erythropoiesis at multiple levels by multiple proteins, RNAs, and chromatin modifications. Blood.

[47] Oburoglu L., Romano M., Taylor N., Kinet S. (2016). Metabolic regulation of hematopoietic stem cell commitment and erythroid differentiation. Curr. Opin. Hematol..

[48] Koury M.J. (2016). Tracking erythroid progenitor cells in times of need and times of plenty. Exp. Hematol..

[49] Haase V.H. (2013). Regulation of erythropoiesis by hypoxia-inducible factors. Blood Rev..

[50] Lee W.-C., Hsu P.-Y., Hsu H.-Y. (2020). Stem cell factor produced by tumor cells expands myeloid-derived suppressor cells in mice. Sci. Rep..

[51] Colmone A., Amorim M., Pontier A.L., Wang S., Jablonski E., Sipkins D.A. (2008). Leukemic cells create bone marrow niches that disrupt the behavior of normal hematopoietic progenitor cells. Science.

[52] Mouchemore K.A., Anderson R.L., Hamilton J.A. (2018). Neutrophils, G-CSF and their contribution to breast cancer metastasis. FEBS J..

[53] Dentelli P., Rosso A., Olgasi C., Camussi G., Brizzi M.F. (2011). IL-3 is a novel target to interfere with tumor vasculature. Oncogene.

[54] Kasper C., Terhaar A., Fosså A., Welt A., Seeber S., Nowrousian M.R. (1997). Recombinant human erythropoietin in the treatment of cancer-related anaemia. Eur. J. Haematol..

[55] Zhang Y., Wei Y., Liu D., Liu F., Li X., Pan L., Pang Y., Chen D. (2017). Role of growth differentiation factor 11 in development, physiology and disease. Oncotarget.

[56] Bashir M., Damineni S., Mukherjee G., Kondaiah P. (2015). Activin-A signaling promotes epithelial–mesenchymal transition, invasion, and metastatic growth of breast cancer. NPJ Breast Cancer.

[57] Wang Y., Gao A., Zhao H., Lu P., Cheng H., Dong F., Gong Y., Ma S., Zheng Y., Zhang H. (2016). Leukemia cell infiltration causes defective erythropoiesis partially through MIP-1α/CCL3. Leukemia.

[58] Liu L., Yu Z., Cheng H., Mao X., Sui W., Deng S., Wei X., Lv J., Du C., Xu J. (2020). Multiple myeloma hinders erythropoiesis and causes anaemia owing to high levels of CCL3 in the bone marrow microenvironment. Sci. Rep..

[59] Silvestris F., Cafforio P., Tucci M., Dammacco F. (2002). Negative regulation of erythroblast maturation by Fas-L^+^/TRAIL^+^ highly malignant plasma cells: A major pathogenetic mechanism of anemia in multiple myeloma. Blood.

[60] Hexner E.O., Serdikoff C., Jan M., Swider C.R., Robinson C., Yang S., Angeles T., Emerson S.G., Carroll M., Ruggeri B. (2008). Lestaurtinib (CEP701) is a JAK2 inhibitor that suppresses JAK2/STAT5 signaling and the proliferation of primary erythroid cells from patients with myeloproliferative disorders. Blood.

[61] Khalil S., Delehanty L., Grado S., Holy M., White Z., Freeman K., Kurita R., Nakamura Y., Bullock G., Goldfarb A. (2017). Iron modulation of erythropoiesis is associated with Scribble-mediated control of the erythropoietin receptor. J. Exp. Med..

[62] Han Y., Liu Q., Hou J., Gu Y., Zhang Y., Chen Z., Fan J., Zhou W., Qiu S., Zhang Y. (2018). Tumor-Induced Generation of Splenic Erythroblast-like Ter-Cells Promotes Tumor Progression. Cell.

[63] Silvestris F., Tucci M., Cafforio P., Dammacco F. (2001). Fas-L up-regulation by highly malignant myeloma plasma cells: Role in the pathogenesis of anemia and disease progression. Blood.

[64] Gilreath J.A., Stenehjem D.D., Rodgers G.M. (2014). Diagnosis and treatment of cancer-related anemia. Am. J. Hematol..

[65] Zowczak M., Iskra M., Torliński L., Cofta S. (2001). Analysis of serum copper and zinc concentrations in cancer patients. Biol. Trace Elem. Res..

[66] Gilreath J.A., Rodgers G.M. (2020). How I treat cancer-associated anemia. Blood.

[67] Pinnix Z.K., Miller L.D., Wang W., D’Agostino R., Kute T., Willingham M.C., Hatcher H., Tesfay L., Sui G., Di X. (2010). Ferroportin and iron regulation in breast cancer progression and prognosis. Sci. Transl. Med..

[68] Vela D., Vela-Gaxha Z. (2018). Differential regulation of hepcidin in cancer and non-cancer tissues and its clinical implications. Exp. Mol. Med..

[69] Mendelson A., Frenette P.S. (2014). Hematopoietic stem cell niche maintenance during homeostasis and regeneration. Nat. Med..

[70] Mei Y., Liu Y., Ji P. (2020). Understanding terminal erythropoiesis: An update on chromatin condensation, enucleation, and reticulocyte maturation. Blood Rev..

[71] Bain B.J. (1996). The bone marrow aspirate of healthy subjects. Br. J. Haematol..

[72] Parmentier S., Kramer M., Weller S., Schuler U., Ordemann R., Rall G., Schaich M., Bornhäuser M., Ehninger G., Kroschinsky F. (2020). Reevaluation of reference values for bone marrow differential counts in 236 healthy bone marrow donors. Ann. Hematol..

[73] Goodman J.W., Hall E.A., Miller K.L., Shinpock S.G. (1985). Interleukin 3 promotes erythroid burst formation in “serum-free” cultures without detectable erythropoietin. Proc. Natl. Acad. Sci. USA.

[74] Emerson S.G., Thomas S., Ferrara J.L., Greenstein J.L. (1989). Developmental regulation of erythropoiesis by hematopoietic growth factors: Analysis on populations of BFU-E from bone marrow, peripheral blood, and fetal liver. Blood.

[75] Muta K., Krantz S.B., Bondurant M.C., Wickrema A. (1994). Distinct roles of erythropoietin, insulin-like growth factor I, and stem cell factor in the development of erythroid progenitor cells. J. Clin. Invest..

[76] De Maria R., Testa U., Luchetti L., Zeuner A., Stassi G., Pelosi E., Riccioni R., Felli N., Samoggia P., Peschle C. (1999). Apoptotic role of Fas/Fas ligand system in the regulation of erythropoiesis. Blood.

[77] Bhoopalan S.V., Huang L.J., Weiss M.J. (2020). Erythropoietin regulation of red blood cell production: From bench to bedside and back. F1000Research.

[78] Muckenthaler M.U., Rivella S., Hentze M.W., Galy B. (2017). A Red Carpet for Iron Metabolism. Cell.

[79] Lee H.-Y., Gao X., Barrasa M.I., Li H., Elmes R.R., Peters L.L., Lodish H.F. (2015). PPAR-α and glucocorticoid receptor synergize to promote erythroid progenitor self-renewal. Nature.

[80] Gutiérrez L., Caballero N., Fernández-Calleja L., Karkoulia E., Strouboulis J. (2020). Regulation of GATA1 levels in erythropoiesis. IUBMB Life.

[81] Pevny L., Simon M.C., Robertson E., Klein W.H., Tsai S.F., D’Agati V., Orkin S.H., Costantini F. (1991). Erythroid differentiation in chimaeric mice blocked by a targeted mutation in the gene for transcription factor GATA-1. Nature.

[82] De Maria R., Zeuner A., Eramo A., Domenichelli C., Bonci D., Grignani F., Srinivasula S.M., Alnemri E.S., Testa U., Peschle C. (1999). Negative regulation of erythropoiesis by caspase-mediated cleavage of GATA-1. Nature.

[83] Han X., Zhang J., Peng Y., Peng M., Chen X., Chen H., Song J., Hu X., Ye M., Li J. (2017). Unexpected role for p19INK4d in posttranscriptional regulation of GATA1 and modulation of human terminal erythropoiesis. Blood.

[84] Ribeil J.-A., Zermati Y., Vandekerckhove J., Cathelin S., Kersual J., Dussiot M., Coulon S., Cruz Moura I., Zeuner A., Kirkegaard-Sørensen T. (2007). Hsp70 regulates erythropoiesis by preventing caspase-3-mediated cleavage of GATA-1. Nature.

[85] Elahi S. (2019). Neglected Cells: Immunomodulatory Roles of CD71^+^ Erythroid Cells. Trends Immunol..

[86] Elahi S., Ertelt J.M., Kinder J.M., Jiang T.T., Zhang X., Xin L., Chaturvedi V., Strong B.S., Qualls J.E., Steinbrecher K.A. (2013). Immunosuppressive CD71^+^ erythroid cells compromise neonatal host defence against infection. Nature.

[87] Shahbaz S., Bozorgmehr N., Koleva P., Namdar A., Jovel J., Fava R.A., Elahi S. (2018). CD71^+^VISTA^+^ erythroid cells promote the development and function of regulatory T cells through TGF-β. PLoS Biol..

[88] Delyea C., Bozorgmehr N., Koleva P., Dunsmore G., Shahbaz S., Huang V., Elahi S. (2018). CD71^+^ Erythroid Suppressor Cells Promote Fetomaternal Tolerance through Arginase-2 and PDL-1. J. Immunol..

[89] Shim Y.A., Weliwitigoda A., Campbell T., Dosanjh M., Johnson P. (2020). Splenic erythroid progenitors decrease TNF-α production by macrophages and reduce systemic inflammation in a mouse model of T cell-induced colitis. Eur. J. Immunol..

[90] Namdar A., Dunsmore G., Shahbaz S., Koleva P., Xu L., Jovel J., Houston S., Elahi S. (2019). CD71^+^ Erythroid Cells Exacerbate HIV-1 Susceptibility, Mediate trans-Infection, and Harbor Infective Viral Particles. mBio.

[91] Bernardes J.P., Mishra N., Tran F., Bahmer T., Best L., Blase J.I., Bordoni D., Franzenburg J., Geisen U., Josephs-Spaulding J. (2020). Longitudinal Multi-omics Analyses Identify Responses of Megakaryocytes, Erythroid Cells, and Plasmablasts as Hallmarks of Severe COVID-19. Immunity.

[92] Grzywa T.M., Sosnowska A., Rydzynska Z., Lazniewski M., Plewczynski D., Klicka K., Malecka M., Rodziewicz-Lurzynska A., Ciepiela O., Justyniarska M. (2021). Potent but transient immunosuppression of T-cells is a general feature of erythroid progenitor cells. bioRxiv.

[93] Dunsmore G., Koleva P., Ghobakhloo N., Sutton R., Ambrosio L., Meng X., Hotte N., Nguyen V., Madsen K.L., Dieleman L.A. (2019). Lower Abundance and Impaired Function of CD71+ Erythroid Cells in Inflammatory Bowel Disease Patients during Pregnancy. J. Crohns Colitis.

[94] Elahi S., Vega-López M.A., Herman-Miguel V., Ramírez-Estudillo C., Mancilla-Ramírez J., Motyka B., West L., Oyegbami O. (2020). CD71^+^ Erythroid Cells in Human Neonates Exhibit Immunosuppressive Properties and Compromise Immune Response Against Systemic Infection in Neonatal Mice. Front. Immunol..

[95] Shahbaz S., Xu L., Osman M., Sligl W., Shields J., Joyce M., Tyrrell L., Oyegbami O., Elahi S. (2020). Erythroid precursors and progenitors suppress adaptive immunity and get invaded by SARS-CoV-2. bioRxiv.

[96] Lindsey Robert B., Sankar S., Michael A., Gayle B., Bernard C.C., Corey C., Brenda C., Erik R.D., Ashley Morris E., Alison G.F. (2016). Prevention and Treatment of Cancer-Related Infections, Version 2.2016, NCCN Clinical Practice Guidelines in Oncology. J. Natl. Compr. Cancer Netw..

[97] Allen B.M., Hiam K.J., Burnett C.E., Venida A., DeBarge R., Tenvooren I., Marquez D.M., Cho N.W., Carmi Y., Spitzer M.H. (2020). Systemic dysfunction and plasticity of the immune macroenvironment in cancer models. Nat. Med..

[98] Wu C., Hua Q., Zheng L. (2020). Generation of Myeloid Cells in Cancer: The Spleen Matters. Front. Immunol..

[99] Strauss L., Guarneri V., Gennari A., Sica A. (2020). Implications of metabolism-driven myeloid dysfunctions in cancer therapy. Cell. Mol. Immunol..

[100] Gillespie M.A., Palii C.G., Sanchez-Taltavull D., Shannon P., Longabaugh W.J.R., Downes D.J., Sivaraman K., Espinoza H.M., Hughes J.R., Price N.D. (2020). Absolute Quantification of Transcription Factors Reveals Principles of Gene Regulation in Erythropoiesis. Mol. Cell.

[101] An X., Schulz V.P., Li J., Wu K., Liu J., Xue F., Hu J., Mohandas N., Gallagher P.G. (2014). Global transcriptome analyses of human and murine terminal erythroid differentiation. Blood.

[102] Li J., Hale J., Bhagia P., Xue F., Chen L., Jaffray J., Yan H., Lane J., Gallagher P.G., Mohandas N. (2014). Isolation and transcriptome analyses of human erythroid progenitors: BFU-E and CFU-E. Blood.

[103] Yan H., Hale J., Jaffray J., Li J., Wang Y., Huang Y., An X., Hillyer C., Wang N., Kinet S. (2018). Developmental differences between neonatal and adult human erythropoiesis. Am. J. Hematol..

[104] Yang Y., Wang H., Chang K.-H., Qu H., Zhang Z., Xiong Q., Qi H., Cui P., Lin Q., Ruan X. (2013). Transcriptome dynamics during human erythroid differentiation and development. Genomics.

[105] Shi L., Lin Y.-H., Sierant M.C., Zhu F., Cui S., Guan Y., Sartor M.A., Tanabe O., Lim K.-C., Engel J.D. (2014). Developmental transcriptome analysis of human erythropoiesis. Hum. Mol. Genet..

[106] Liu X., Zhang Y., Ni M., Cao H., Signer R.A.J., Li D., Li M., Gu Z., Hu Z., Dickerson K.E. (2017). Regulation of mitochondrial biogenesis in erythropoiesis by mTORC1-mediated protein translation. Nat. Cell Biol..

[107] Huang P., Zhao Y., Zhong J., Zhang X., Liu Q., Qiu X., Chen S., Yan H., Hillyer C., Mohandas N. (2020). Putative regulators for the continuum of erythroid differentiation revealed by single-cell transcriptome of human BM and UCB cells. Proc. Natl. Acad. Sci. USA.

[108] Gautier E.-F., Ducamp S., Leduc M., Salnot V., Guillonneau F., Dussiot M., Hale J., Giarratana M.-C., Raimbault A., Douay L. (2016). Comprehensive Proteomic Analysis of Human Erythropoiesis. Cell Rep..

[109] Amon S., Meier-Abt F., Gillet L.C., Dimitrieva S., Theocharides A.P.A., Manz M.G., Aebersold R. (2019). Sensitive Quantitative Proteomics of Human Hematopoietic Stem and Progenitor Cells by Data-independent Acquisition Mass Spectrometry. Mol. Cell Proteom..

[110] Brand M., Ranish J.A., Kummer N.T., Hamilton J., Igarashi K., Francastel C., Chi T.H., Crabtree G.R., Aebersold R., Groudine M. (2004). Dynamic changes in transcription factor complexes during erythroid differentiation revealed by quantitative proteomics. Nat. Struct. Mol. Biol..

[111] Jassinskaja M., Johansson E., Kristiansen T.A., Åkerstrand H., Sjöholm K., Hauri S., Malmström J., Yuan J., Hansson J. (2017). Comprehensive Proteomic Characterization of Ontogenic Changes in Hematopoietic Stem and Progenitor Cells. Cell Rep..

[112] Mello F.V., Land M.G.P., Costa E.S., Teodósio C., Sanchez M.-L., Bárcena P., Peres R.T., Pedreira C.E., Alves L.R., Orfao A. (2019). Maturation-associated gene expression profiles during normal human bone marrow erythropoiesis. Cell Death Discov..

[113] Panday A., Sahoo M.K., Osorio D., Batra S. (2015). NADPH oxidases: An overview from structure to innate immunity-associated pathologies. Cell. Mol. Immunol..

[114] Franchina D.G., Dostert C., Brenner D. (2018). Reactive Oxygen Species: Involvement in T Cell Signaling and Metabolism. Trends Immunol..

[115] Ohl K., Tenbrock K. (2018). Reactive Oxygen Species as Regulators of MDSC-Mediated Immune Suppression. Front. Immunol..

[116] Xia W., Sainan Y., Xin P., Silian H., Zailin Y., Xiaomin S., Wen C., Yong Z. (2020). CD45+ Erythroid Progenitor Cell Contribute to Antiangiogenic Drug Resistance Through Reactive Oxygen Species in Lymphoma. Res. Sq..

[117] Kotsafti A., Scarpa M., Castagliuolo I., Scarpa M. (2020). Reactive Oxygen Species and Antitumor Immunity-From Surveillance to Evasion. Cancers.

[118] Lewis S.M., Williams A., Eisenbarth S.C. (2019). Structure and function of the immune system in the spleen. Sci. Immunol..

[119] Imai T., Ishida H., Suzue K., Taniguchi T., Okada H., Shimokawa C., Hisaeda H. (2015). Cytotoxic activities of CD8^+^ T cells collaborate with macrophages to protect against blood-stage murine malaria. Elife.

[120] Ilieva M., Nielsen J., Korshunova I., Gotfryd K., Bock E., Pankratova S., Michel T.M. (2019). Artemin and an Artemin-Derived Peptide, Artefin, Induce Neuronal Survival, and Differentiation Through Ret and NCAM. Front. Mol. Neurosci..

[121] Wang J., Wang H., Cai J., Du S., Xin B., Wei W., Zhang T., Shen X. (2018). Artemin regulates CXCR4 expression to induce migration and invasion in pancreatic cancer cells through activation of NF-κB signaling. Exp. Cell Res..

[122] Song Z., Yang F., Du H., Li X., Liu J., Dong M., Xu X. (2018). Role of artemin in non-small cell lung cancer. Thorac. Cancer.

[123] Li T.-J., Li H., Zhang W.-H., Xu S.-S., Jiang W., Li S., Gao H.-L., Han X., Xu H.-X., Wu C.-T. (2020). Human splenic TER cells: A relevant prognostic factor acting via the artemin-GFRα3-ERK pathway in pancreatic ductal adenocarcinoma. Int. J. Cancer.

[124] Dzierzak E., Philipsen S. (2013). Erythropoiesis: Development and differentiation. Cold Spring Harb. Perspect. Med..

[125] Ileana C., Sjaak P. (2014). Flicking the switch: Adult hemoglobin expression in erythroid cells derived from cord blood and human induced pluripotent stem cells. Haematologica.

[126] Baron M.H., Isern J., Fraser S.T. (2012). The embryonic origins of erythropoiesis in mammals. Blood.

[127] Riley R.S., Ben-Ezra J.M., Goel R., Tidwell A. (2001). Reticulocytes and reticulocyte enumeration. J. Clin. Lab. Anal..

[128] Paulson R.F., Hariharan S., Little J.A. (2020). Stress erythropoiesis: Definitions and models for its study. Exp. Hematol..

[129] Paulson R.F., Ruan B., Hao S., Chen Y. (2020). Stress Erythropoiesis is a Key Inflammatory Response. Cells.

[130] Jackson A., Nanton M.R., O’Donnell H., Akue A.D., McSorley S.J. (2010). Innate immune activation during Salmonella infection initiates extramedullary erythropoiesis and splenomegaly. J. Immunol..

[131] Rinchai D., Altman M.C., Konza O., Hässler S., Martina F., Toufiq M., Garand M., Kabeer B.S.A., Palucka K., Mejias A. (2020). Definition of erythroid cell-positive blood transcriptome phenotypes associated with severe respiratory syncytial virus infection. Clin. Transl. Med..

[132] Tabares Calvache E., Tabares Calvache A.D., Faulhaber G.A.M. (2020). Systematic review about etiologic association to the leukoerythroblastic reaction. Int. J. Lab. Hematol..

[133] Delsol G., Guiu-Godfrin B., Guiu M., Pris J., Corberand J., Fabre J. (1979). Leukoerythroblastosis and cancer frequency, prognosis, and physiopathologic significance. Cancer.

[134] Kornblau S.M., Cohen A.C., Soper D., Huang Y.-W., Cesano A. (2014). Age-related changes of healthy bone marrow cell signaling in response to growth factors provide insight into low risk MDS. Cytom. Part B Clin. Cytom..

[135] Oikonomidou P.R., Rivella S. (2018). What can we learn from ineffective erythropoiesis in thalassemia?. Blood Rev..

[136] Manso B.A., Zhang H., Mikkelson M.G., Gwin K.A., Secreto C.R., Ding W., Parikh S.A., Kay N.E., Medina K.L. (2019). Bone marrow hematopoietic dysfunction in untreated chronic lymphocytic leukemia patients. Leukemia.

[137] Mussai F., De Santo C., Abu-Dayyeh I., Booth S., Quek L., McEwen-Smith R.M., Qureshi A., Dazzi F., Vyas P., Cerundolo V. (2013). Acute myeloid leukemia creates an arginase-dependent immunosuppressive microenvironment. Blood.

[138] Stefanie G., Manuel R.-P., Paul J., Annemarie K., Felix B., Julian G., Christoph Z., Ulrich G., Guido K., Frank L. (2018). Transforming growth factor β1-mediated functional inhibition of mesenchymal stromal cells in myelodysplastic syndromes and acute myeloid leukemia. Haematologica.

[139] Gong Y., Zhao M., Yang W., Gao A., Yin X., Hu L., Wang X., Xu J., Hao S., Cheng T. (2018). Megakaryocyte-derived excessive transforming growth factor β1 inhibits proliferation of normal hematopoietic stem cells in acute myeloid leukemia. Exp. Hematol..

[140] Bruns I., Cadeddu R.-P., Brueckmann I., Fröbel J., Geyh S., Büst S., Fischer J.C., Roels F., Wilk C.M., Schildberg F.A. (2012). Multiple myeloma–related deregulation of bone marrow–derived CD34^+^ hematopoietic stem and progenitor cells. Blood.

[141] Waclawiczek A., Hamilton A., Rouault-Pierre K., Abarrategi A., Albornoz M.G., Miraki-Moud F., Bah N., Gribben J., Fitzgibbon J., Taussig D. (2020). Mesenchymal niche remodeling impairs hematopoiesis via stanniocalcin 1 in acute myeloid leukemia. J. Clin. Invest..

[142] Wu W.-C., Sun H.-W., Chen H.-T., Liang J., Yu X.-J., Wu C., Wang Z., Zheng L. (2014). Circulating hematopoietic stem and progenitor cells are myeloid-biased in cancer patients. Proc. Natl. Acad. Sci. USA.

[143] Al Sayed M.F., Amrein M.A., Bührer E.D., Huguenin A.-L., Radpour R., Riether C., Ochsenbein A.F. (2019). T-cell–Secreted TNFα Induces Emergency Myelopoiesis and Myeloid-Derived Suppressor Cell Differentiation in Cancer. Cancer Res..

[144] Wu C., Ning H., Liu M., Lin J., Luo S., Zhu W., Xu J., Wu W.-C., Liang J., Shao C.-K. (2018). Spleen mediates a distinct hematopoietic progenitor response supporting tumor-promoting myelopoiesis. J. Clin. Invest..

[145] Engblom C., Pfirschke C., Pittet M.J. (2016). The role of myeloid cells in cancer therapies. Nat. Rev. Cancer.

[146] Giles A.J., Reid C.M., Evans J.D., Murgai M., Vicioso Y., Highfill S.L., Kasai M., Vahdat L., Mackall C.L., Lyden D. (2016). Activation of Hematopoietic Stem/Progenitor Cells Promotes Immunosuppression Within the Pre–metastatic Niche. Cancer Res..

[147] Sugiyama T., Kohara H., Noda M., Nagasawa T. (2006). Maintenance of the hematopoietic stem cell pool by CXCL12-CXCR4 chemokine signaling in bone marrow stromal cell niches. Immunity.

[148] Glait-Santar C., Desmond R., Feng X., Bat T., Chen J., Heuston E., Mizukawa B., Mulloy J.C., Bodine D.M., Larochelle A. (2015). Functional Niche Competition Between Normal Hematopoietic Stem and Progenitor Cells and Myeloid Leukemia Cells. Stem Cells.

[149] O’Donnell R.K., Falcon B., Hanson J., Goldstein W.E., Perruzzi C., Rafii S., Aird W.C., Benjamin L.E. (2016). VEGF-A/VEGFR Inhibition Restores Hematopoietic Homeostasis in the Bone Marrow and Attenuates Tumor Growth. Cancer Res..

[150] Peinado H., Alečković M., Lavotshkin S., Matei I., Costa-Silva B., Moreno-Bueno G., Hergueta-Redondo M., Williams C., García-Santos G., Ghajar C. (2012). Melanoma exosomes educate bone marrow progenitor cells toward a pro-metastatic phenotype through MET. Nat. Med..

[151] Li X., Wang S., Zhu R., Li H., Han Q., Zhao R.C. (2016). Lung tumor exosomes induce a pro-inflammatory phenotype in mesenchymal stem cells via NFκB-TLR signaling pathway. J. Hematol. Oncol..

[152] Zhang B., Ho Y.W., Huang Q., Maeda T., Lin A., Lee S.U., Hair A., Holyoake T.L., Huettner C., Bhatia R. (2012). Altered microenvironmental regulation of leukemic and normal stem cells in chronic myelogenous leukemia. Cancer Cell.

[153] Lopes M., Duarte T.L., Teles M.J., Mosteo L., Chacim S., Aguiar E., Pereira-Reis J., Oliveira M., Silva A.M.N., Gonçalves N. (2020). Loss of erythroblasts in acute myeloid leukemia causes iron redistribution with clinical implications. bioRxiv.

[154] Cheng H., Hao S., Liu Y., Pang Y., Ma S., Dong F., Xu J., Zheng G., Li S., Yuan W. (2015). Leukemic marrow infiltration reveals a novel role for Egr3 as a potent inhibitor of normal hematopoietic stem cell proliferation. Blood.

[155] Bouchnita A., Eymard N., Moyo T.K., Koury M.J., Volpert V. (2016). Bone marrow infiltration by multiple myeloma causes anemia by reversible disruption of erythropoiesis. Am. J. Hematol..

[156] Haase V.H. (2010). Hypoxic regulation of erythropoiesis and iron metabolism. Am. J. Physiol. Ren. Physiol..

[157] Dev A., Fang J., Sathyanarayana P., Pradeep A., Emerson C., Wojchowski D.M. (2010). During EPO or anemia challenge, erythroid progenitor cells transit through a selectively expandable proerythroblast pool. Blood.

[158] Jelkmann W. (2011). Regulation of erythropoietin production. J. Physiol..

[159] Viallard C., Larrivée B. (2017). Tumor angiogenesis and vascular normalization: Alternative therapeutic targets. Angiogenesis.

[160] Yang J., Yan J., Liu B. (2018). Targeting VEGF/VEGFR to Modulate Antitumor Immunity. Front. Immunol..

[161] Kut C., Mac Gabhann F., Popel A.S. (2007). Where is VEGF in the body? A meta-analysis of VEGF distribution in cancer. Br. J. Cancer.

[162] Xue Y., Lim S., Yang Y., Wang Z., Jensen L.D.E., Hedlund E.-M., Andersson P., Sasahara M., Larsson O., Galter D. (2012). PDGF-BB modulates hematopoiesis and tumor angiogenesis by inducing erythropoietin production in stromal cells. Nat. Med..

[163] Greenwald A.C., Licht T., Kumar S., Oladipupo S.S., Iyer S., Grunewald M., Keshet E. (2019). VEGF expands erythropoiesis via hypoxia-independent induction of erythropoietin in noncanonical perivascular stromal cells. J. Exp. Med..

[164] Liu Y., Pop R., Sadegh C., Brugnara C., Haase V.H., Socolovsky M. (2006). Suppression of Fas-FasL coexpression by erythropoietin mediates erythroblast expansion during the erythropoietic stress response in vivo. Blood.

[165] Bordini J., Bertilaccio M.T., Ponzoni M., Fermo I., Chesi M., Bergsagel P.L., Camaschella C., Campanella A. (2015). Erythroblast apoptosis and microenvironmental iron restriction trigger anemia in the VK*MYC model of multiple myeloma. Haematologica.

[166] Testa U. (2004). Apoptotic mechanisms in the control of erythropoiesis. Leukemia.

[167] Gregory T., Yu C., Ma A., Orkin S.H., Blobel G.A., Weiss M.J. (1999). GATA-1 and erythropoietin cooperate to promote erythroid cell survival by regulating bcl-xL expression. Blood.

[168] Tanaka H., Matsumura I., Nakajima K., Daino H., Sonoyama J., Yoshida H., Oritani K., Machii T., Yamamoto M., Hirano T. (2000). GATA-1 blocks IL-6-induced macrophage differentiation and apoptosis through the sustained expression of cyclin D1 and bcl-2 in a murine myeloid cell line M1. Blood.

[169] Spierings D.C.J., de Vries E.G.E., Timens W., Groen H.J.M., Boezen H.M., de Jong S. (2003). Expression of TRAIL and TRAIL Death Receptors in Stage III Non-Small Cell Lung Cancer Tumors. Clin. Cancer Res..

[170] Secchiero P., Melloni E., Heikinheimo M., Mannisto S., Di Pietro R., Iacone A., Zauli G. (2004). TRAIL regulates normal erythroid maturation through an ERK-dependent pathway. Blood.

[171] Zamai L., Secchiero P., Pierpaoli S., Bassini A., Papa S., Alnemri E.S., Guidotti L., Vitale M., Zauli G. (2000). TNF-related apoptosis-inducing ligand (TRAIL) as a negative regulator of normal human erythropoiesis. Blood.

[172] Batlle E., Massagué J. (2019). Transforming Growth Factor-β Signaling in Immunity and Cancer. Immunity.

[173] González-Santiago A.E., Mendoza-Topete L.A., Sánchez-Llamas F., Troyo-Sanromán R., Gurrola-Díaz C.M. (2011). TGF-β1 serum concentration as a complementary diagnostic biomarker of lung cancer: Establishment of a cut-point value. J. Clin. Lab. Anal..

[174] Shirai Y., Kawata S., Tamura S., Ito N., Tsushima H., Takaishi K., Kiso S., Matsuzawa Y. (1994). Plasma transforming growth factor-beta 1 in patients with hepatocellular carcinoma. Comparison with chronic liver diseases. Cancer.

[175] Zermati Y., Fichelson S., Valensi F., Freyssinier J.M., Rouyer-Fessard P., Cramer E., Guichard J., Varet B., Hermine O. (2000). Transforming growth factor inhibits erythropoiesis by blocking proliferation and accelerating differentiation of erythroid progenitors. Exp. Hematol..

[176] Kuhikar R., Khan N., Philip J., Melinkeri S., Kale V., Limaye L. (2020). Transforming growth factor β1 accelerates and enhances in vitro red blood cell formation from hematopoietic stem cells by stimulating mitophagy. Stem Cell Res. Ther..

[177] Akel S., Petrow-Sadowski C., Laughlin M.J., Ruscetti F.W. (2003). Neutralization of autocrine transforming growth factor-beta in human cord blood CD34^+^CD38^−^Lin^−^ cells promotes stem-cell-factor-mediated erythropoietin-independent early erythroid progenitor development and reduces terminal differentiation. Stem Cells.

[178] Gao X., Lee H.Y., da Rocha E.L., Zhang C., Lu Y.F., Li D., Feng Y., Ezike J., Elmes R.R., Barrasa M.I. (2016). TGF-β inhibitors stimulate red blood cell production by enhancing self-renewal of BFU-E erythroid progenitors. Blood.

[179] Taniguchi S., Elhance A., Van Duzer A., Kumar S., Leitenberger J.J., Oshimori N. (2020). Tumor-initiating cells establish an IL-33–TGF-β niche signaling loop to promote cancer progression. Science.

[180] Fournié J.-J., Poupot M. (2018). The Pro-tumorigenic IL-33 Involved in Antitumor Immunity: A Yin and Yang Cytokine. Front. Immunol..

[181] Swann J.W., Koneva L.A., Regan-Komito D., Sansom S.N., Powrie F., Griseri T. (2020). IL-33 promotes anemia during chronic inflammation by inhibiting differentiation of erythroid progenitors. J. Exp. Med..

[182] Suragani R.N.V.S., Cadena S.M., Cawley S.M., Sako D., Mitchell D., Li R., Davies M.V., Alexander M.J., Devine M., Loveday K.S. (2014). Transforming growth factor-β superfamily ligand trap ACE-536 corrects anemia by promoting late-stage erythropoiesis. Nat. Med..

[183] Martinez P.A., Li R., Ramanathan H.N., Bhasin M., Pearsall R.S., Kumar R., Suragani R.N.V.S. (2020). Smad2/3-pathway ligand trap luspatercept enhances erythroid differentiation in murine β-thalassaemia by increasing GATA-1 availability. J. Cell. Mol. Med..

[184] Dussiot M., Maciel T.T., Fricot A., Chartier C., Negre O., Veiga J., Grapton D., Paubelle E., Payen E., Beuzard Y. (2014). An activin receptor IIA ligand trap corrects ineffective erythropoiesis in β-thalassemia. Nat. Med..

[185] Han Y., Wang H., Shao Z. (2015). GDF11 Increased in Patients with Myelodysplastic Syndrome. Blood.

[186] Tanno T., Bhanu N.V., Oneal P.A., Goh S.H., Staker P., Lee Y.T., Moroney J.W., Reed C.H., Luban N.L., Wang R.H. (2007). High levels of GDF15 in thalassemia suppress expression of the iron regulatory protein hepcidin. Nat. Med..

[187] Lenox L.E., Perry J.M., Paulson R.F. (2005). BMP4 and Madh5 regulate the erythroid response to acute anemia. Blood.

[188] Perry J.M., Harandi O.F., Paulson R.F. (2007). BMP4, SCF, and hypoxia cooperatively regulate the expansion of murine stress erythroid progenitors. Blood.

[189] Kim A., Nemeth E. (2015). New insights into iron regulation and erythropoiesis. Curr. Opin. Hematol..

[190] Clara C., Antonella N., Laura S. (2020). Iron metabolism and iron disorders revisited in the hepcidin era. Haematologica.

[191] Ludwig H., Müldür E., Endler G., Hübl W. (2013). Prevalence of iron deficiency across different tumors and its association with poor performance status, disease status and anemia. Ann. Oncol..

[192] Liu S., Bhattacharya S., Han A., Suragani R.N., Zhao W., Fry R.C., Chen J.J. (2008). Haem-regulated eIF2alpha kinase is necessary for adaptive gene expression in erythroid precursors under the stress of iron deficiency. Br. J. Haematol..

[193] Masahiro K., Hiroki K., Hiroshi H., Ari I.-N., Tohru F., Akihiko M., Yukihiro I., Kenji I., Wataru H., Naohisa T. (2017). Iron-heme-Bach1 axis is involved in erythroblast adaptation to iron deficiency. Haematologica.

[194] Bullock G.C., Delehanty L.L., Talbot A.-L., Gonias S.L., Tong W.-H., Rouault T.A., Dewar B., Macdonald J.M., Chruma J.J., Goldfarb A.N. (2010). Iron control of erythroid development by a novel aconitase-associated regulatory pathway. Blood.

[195] Shaw G.C., Cope J.J., Li L., Corson K., Hersey C., Ackermann G.E., Gwynn B., Lambert A.J., Wingert R.A., Traver D. (2006). Mitoferrin is essential for erythroid iron assimilation. Nature.

[196] Schranzhofer M., Schifrer M., Cabrera J.A., Kopp S., Chiba P., Beug H., Müllner E.W. (2006). Remodeling the regulation of iron metabolism during erythroid differentiation to ensure efficient heme biosynthesis. Blood.

[197] Brown R.A.M., Richardson K.L., Kabir T.D., Trinder D., Ganss R., Leedman P.J. (2020). Altered Iron Metabolism and Impact in Cancer Biology, Metastasis, and Immunology. Front. Oncol..

[198] Pfeifhofer-Obermair C., Tymoszuk P., Petzer V., Weiss G., Nairz M. (2018). Iron in the Tumor Microenvironment—Connecting the Dots. Front. Oncol..

[199] Daniels T.R., Delgado T., Rodriguez J.A., Helguera G., Penichet M.L. (2006). The transferrin receptor part I: Biology and targeting with cytotoxic antibodies for the treatment of cancer. Clin. Immunol..

[200] Ganz T. (2019). Anemia of Inflammation. N. Engl. J. Med..

[201] Weiss G., Ganz T., Goodnough L.T. (2019). Anemia of inflammation. Blood.

[202] Zheng D., Liwinski T., Elinav E. (2020). Inflammasome activation and regulation: Toward a better understanding of complex mechanisms. Cell Discov..

[203] Tyrkalska S.D., Pérez-Oliva A.B., Rodríguez-Ruiz L., Martínez-Morcillo F.J., Alcaraz-Pérez F., Martínez-Navarro F.J., Lachaud C., Ahmed N., Schroeder T., Pardo-Sánchez I. (2019). Inflammasome Regulates Hematopoiesis through Cleavage of the Master Erythroid Transcription Factor GATA1. Immunity.

[204] Hamarsheh S., Osswald L., Saller B.S., Unger S., De Feo D., Vinnakota J.M., Konantz M., Uhl F.M., Becker H., Lübbert M. (2020). Oncogenic KrasG12D causes myeloproliferation via NLRP3 inflammasome activation. Nat. Commun..

[205] Prince O.D., Langdon J.M., Layman A.J., Prince I.C., Sabogal M., Mak H.H., Berger A.E., Cheadle C., Chrest F.J., Yu Q. (2012). Late stage erythroid precursor production is impaired in mice with chronic inflammation. Haematologica.

[206] Ivashkiv L.B. (2018). IFNγ: Signalling, epigenetics and roles in immunity, metabolism, disease and cancer immunotherapy. Nat. Rev. Immunol..

[207] Libregts S.F., Gutiérrez L., de Bruin A.M., Wensveen F.M., Papadopoulos P., van Ijcken W., Ozgür Z., Philipsen S., Nolte M.A. (2011). Chronic IFN-γ production in mice induces anemia by reducing erythrocyte life span and inhibiting erythropoiesis through an IRF-1/PU.1 axis. Blood.

[208] Dai C., Krantz S.B. (1999). Interferon γ Induces Upregulation and Activation of Caspases 1, 3, and 8 to Produce Apoptosis in Human Erythroid Progenitor Cells. Blood.

[209] Wang C.Q., Udupa K.B., Lipschitz D.A. (1995). Interferon-γ exerts its negative regulatory effect primarily on the earliest stages of murine erythroid progenitor cell development. J. Cell. Physiol..

[210] Dai C.-H., Price J.O., Brunner T., Krantz S.B. (1998). Fas Ligand Is Present in Human Erythroid Colony-Forming Cells and Interacts with Fas Induced by Interferon γ to Produce Erythroid Cell Apoptosis. Blood.

[211] Zhang P., Zhang X., Iwama A., Yu C., Smith K.A., Mueller B.U., Narravula S., Torbett B.E., Orkin S.H., Tenen D.G. (2000). PU. 1 inhibits GATA-1 function and erythroid differentiation by blocking GATA-1 DNA binding. Blood.

[212] Yamada T., Kondoh N., Matsumoto M., Yoshida M., Maekawa A., Oikawa T. (1997). Overexpression of PU.1 induces growth and differentiation inhibition and apoptotic cell death in murine erythroleukemia cells. Blood.

[213] Burda P., Laslo P., Stopka T. (2010). The role of PU.1 and GATA-1 transcription factors during normal and leukemogenic hematopoiesis. Leukemia.

[214] Montfort A., Colacios C., Levade T., Andrieu-Abadie N., Meyer N., Ségui B. (2019). The TNF Paradox in Cancer Progression and Immunotherapy. Front. Immunol..

[215] Rusten L.S., Jacobsen S.E. (1995). Tumor necrosis factor (TNF)-alpha directly inhibits human erythropoiesis in vitro: Role of p55 and p75 TNF receptors. Blood.

[216] Tsopra O.A., Ziros P.G., Lagadinou E.D., Symeonidis A., Kouraklis-Symeonidis A., Thanopoulou E., Angelopoulou M.K., Vassilakopoulos T.P., Pangalis G.A., Zoumbos N.C. (2009). Disease-related anemia in chronic lymphocytic leukemia is not due to intrinsic defects of erythroid precursors: A possible pathogenetic role for tumor necrosis factor-alpha. Acta Haematol..

[217] Papadaki H.A., Kritikos H.D., Valatas V., Boumpas D.T., Eliopoulos G.D. (2002). Anemia of chronic disease in rheumatoid arthritis is associated with increased apoptosis of bone marrow erythroid cells: Improvement following anti–tumor necrosis factor-α antibody therapy. Blood.

[218] Jacobs-Helber S.M., Roh K.-H., Bailey D., Dessypris E.N., Ryan J.J., Chen J., Wickrema A., Barber D.L., Dent P., Sawyer S.T. (2003). Tumor necrosis factor-alpha expressed constitutively in erythroid cells or induced by erythropoietin has negative and stimulatory roles in normal erythropoiesis and erythroleukemia. Blood.

[219] Bibikova E., Youn M.-Y., Danilova N., Ono-Uruga Y., Konto-Ghiorghi Y., Ochoa R., Narla A., Glader B., Lin S., Sakamoto K.M. (2014). TNF-mediated inflammation represses GATA1 and activates p38 MAP kinase in RPS19-deficient hematopoietic progenitors. Blood.

[220] Hernandez-Hernandez A., Ray P., Litos G., Ciro M., Ottolenghi S., Beug H., Boyes J. (2006). Acetylation and MAPK phosphorylation cooperate to regulate the degradation of active GATA-1. EMBO J..

[221] Manso B.A., Krull J.E., Gwin K.A., Lothert P.K., Welch B.M., Novak A.J., Parikh S.A., Kay N.E., Medina K.L. (2021). Chronic lymphocytic leukemia B-cell-derived TNFα impairs bone marrow myelopoiesis. iScience.

[222] Schooley J.C., Kullgren B., Allison A.C. (1987). Inhibition by interleukin-1 of the action of erythropoietin on erythroid precursors and its possible role in the pathogenesis of hypoplastic anaemias. Br. J. Haematol..

[223] Chou D.B., Sworder B., Bouladoux N., Roy C.N., Uchida A.M., Grigg M., Robey P.G., Belkaid Y. (2012). Stromal-derived IL-6 alters the balance of myeloerythroid progenitors during Toxoplasma gondii infection. J. Leukoc. Biol..

[224] Gołab J., Zagozdzon R., Stokłosa T., Jakóbisiak M., Pojda Z., Machaj E. (1998). Erythropoietin prevents the development of interleukin-12-induced anemia and thrombocytopenia but does not decrease its antitumor activity in mice. Blood.

[225] De la Fuente López M., Landskron G., Parada D., Dubois-Camacho K., Simian D., Martinez M., Romero D., Roa J.C., Chahuán I., Gutiérrez R. (2018). The relationship between chemokines CCL2, CCL3, and CCL4 with the tumor microenvironment and tumor-associated macrophage markers in colorectal cancer. Tumor Biol..

[226] Buck I., Morceau F., Cristofanon S., Heintz C., Chateauvieux S., Reuter S., Dicato M., Diederich M. (2008). Tumor necrosis factor α inhibits erythroid differentiation in human erythropoietin-dependent cells involving p38 MAPK pathway, GATA-1 and FOG-1 downregulation and GATA-2 upregulation. Biochem. Pharmacol..

[227] Liao C., Prabhu K.S., Paulson R.F. (2018). Monocyte-derived macrophages expand the murine stress erythropoietic niche during the recovery from anemia. Blood.

[228] Millot S., Andrieu V., Letteron P., Lyoumi S., Hurtado-Nedelec M., Karim Z., Thibaudeau O., Bennada S., Charrier J.L., Lasocki S. (2010). Erythropoietin stimulates spleen BMP4-dependent stress erythropoiesis and partially corrects anemia in a mouse model of generalized inflammation. Blood.

[229] Bennett L.F., Liao C., Quickel M.D., Yeoh B.S., Vijay-Kumar M., Hankey-Giblin P., Prabhu K.S., Paulson R.F. (2019). Inflammation induces stress erythropoiesis through heme-dependent activation of SPI-C. Sci. Signal..

[230] Corazza F., Beguin Y., Bergmann P., André M., Ferster A., Devalck C., Fondu P., Buyse M., Sariban E. (1998). Anemia in children with cancer is associated with decreased erythropoietic activity and not with inadequate erythropoietin production. Blood.

[231] Chen Y., Xiang J., Qian F., Diwakar B.T., Ruan B., Hao S., Prabhu K.S., Paulson R.F. (2020). Epo receptor signaling in macrophages alters the splenic niche to promote erythroid differentiation. Blood.

[232] Song X., Krelin Y., Dvorkin T., Bjorkdahl O., Segal S., Dinarello C.A., Voronov E., Apte R.N. (2005). CD11b^+^/Gr-1^+^ Immature Myeloid Cells Mediate Suppression of T Cells in Mice Bearing Tumors of IL-1β-Secreting Cells. J. Immunol..

[233] Jing W., Guo X., Qin F., Li Y., Wang G., Bi Y., Jin X., Han L., Dong X., Zhao Y. (2021). G-CSF shifts erythropoiesis from bone marrow into spleen in the setting of systemic inflammation. Life Sci. Alliance.

[234] McKim D.B., Yin W., Wang Y., Cole S.W., Godbout J.P., Sheridan J.F. (2018). Social Stress Mobilizes Hematopoietic Stem Cells to Establish Persistent Splenic Myelopoiesis. Cell Rep..

[235] Alamo I.G., Kannan K.B., Loftus T.J., Ramos H., Efron P.A., Mohr A.M. (2017). Severe trauma and chronic stress activates extramedullary erythropoiesis. J. Trauma Acute Care Surg..

[236] Zeuner A., Pedini F., Signore M., Testa U., Pelosi E., Peschle C., De Maria R. (2003). Stem cell factor protects erythroid precursor cells from chemotherapeutic agents via up-regulation of BCL-2 family proteins. Blood.

[237] Bartucci M., Dattilo R., Martinetti D., Todaro M., Zapparelli G., Di Virgilio A., Biffoni M., De Maria R., Zeuner A. (2011). Prevention of Chemotherapy-Induced Anemia and Thrombocytopenia by Constant Administration of Stem Cell Factor. Clin. Cancer Res..

[238] Gao P., Zhang H., Dinavahi R., Li F., Xiang Y., Raman V., Bhujwalla Z.M., Felsher D.W., Cheng L., Pevsner J. (2007). HIF-dependent antitumorigenic effect of antioxidants in vivo. Cancer Cell.

[239] Le Gal K., Ibrahim M.X., Wiel C., Sayin V.I., Akula M.K., Karlsson C., Dalin M.G., Akyürek L.M., Lindahl P., Nilsson J. (2015). Antioxidants can increase melanoma metastasis in mice. Sci. Transl. Med..

[240] Sayin V.I., Ibrahim M.X., Larsson E., Nilsson J.A., Lindahl P., Bergo M.O. (2014). Antioxidants Accelerate Lung Cancer Progression in Mice. Sci. Transl. Med..

[241] Reczek C.R., Chandel N.S. (2017). The Two Faces of Reactive Oxygen Species in Cancer. Annu. Rev. Cancer Biol..

[242] Firczuk M., Bajor M., Graczyk-Jarzynka A., Fidyt K., Goral A., Zagozdzon R. (2020). Harnessing altered oxidative metabolism in cancer by augmented prooxidant therapy. Cancer Lett..

[243] Naing A., Infante J.R., Papadopoulos K.P., Chan I.H., Shen C., Ratti N.P., Rojo B., Autio K.A., Wong D.J., Patel M.R. (2018). PEGylated IL-10 (Pegilodecakin) Induces Systemic Immune Activation, CD8^+^ T Cell Invigoration and Polyclonal T Cell Expansion in Cancer Patients. Cancer Cell.

[244] Vahl J.M., Friedrich J., Mittler S., Trump S., Heim L., Kachler K., Balabko L., Fuhrich N., Geppert C.-I., Trufa D.I. (2017). Interleukin-10-regulated tumour tolerance in non-small cell lung cancer. Br. J. Cancer.

[245] Qiao J., Liu Z., Dong C., Luan Y., Zhang A., Moore C., Fu K., Peng J., Wang Y., Ren Z. (2019). Targeting Tumors with IL-10 Prevents Dendritic Cell-Mediated CD8^+^ T Cell Apoptosis. Cancer Cell.

[246] Oft M. (2014). IL-10: Master switch from tumor-promoting inflammation to antitumor immunity. Cancer Immunol. Res..

[247] Mumm J.B., Emmerich J., Zhang X., Chan I., Wu L., Mauze S., Blaisdell S., Basham B., Dai J., Grein J. (2011). IL-10 elicits IFNγ-dependent tumor immune surveillance. Cancer Cell.

[248] Oft M. (2019). Immune regulation and cytotoxic T cell activation of IL-10 agonists—Preclinical and clinical experience. Semin. Immunol..

[249] Sun C., Mezzadra R., Schumacher T.N. (2018). Regulation and Function of the PD-L1 Checkpoint. Immunity.

[250] Gong J., Chehrazi-Raffle A., Reddi S., Salgia R. (2018). Development of PD-1 and PD-L1 inhibitors as a form of cancer immunotherapy: A comprehensive review of registration trials and future considerations. J. Immunother. Cancer.

[251] Chamoto K., Hatae R., Honjo T. (2020). Current issues and perspectives in PD-1 blockade cancer immunotherapy. Int. J. Clin. Oncol..

[252] Mariathasan S., Turley S.J., Nickles D., Castiglioni A., Yuen K., Wang Y., Kadel E.E., Koeppen H., Astarita J.L., Cubas R. (2018). TGFβ attenuates tumour response to PD-L1 blockade by contributing to exclusion of T cells. Nature.

[253] Li S., Liu M., Do M.H., Chou C., Stamatiades E.G., Nixon B.G., Shi W., Zhang X., Li P., Gao S. (2020). Cancer immunotherapy via targeted TGF-β signalling blockade in TH cells. Nature.

[254] Tauriello D.V.F., Palomo-Ponce S., Stork D., Berenguer-Llergo A., Badia-Ramentol J., Iglesias M., Sevillano M., Ibiza S., Cañellas A., Hernando-Momblona X. (2018). TGFβ drives immune evasion in genetically reconstituted colon cancer metastasis. Nature.

[255] Groeneveldt C., van Hall T., van der Burg S.H., Ten Dijke P., van Montfoort N. (2020). Immunotherapeutic Potential of TGF-β Inhibition and Oncolytic Viruses. Trends Immunol..

[256] DeBerry J.J., Saloman J.L., Dragoo B.K., Albers K.M., Davis B.M. (2015). Artemin Immunotherapy Is Effective in Preventing and Reversing Cystitis-Induced Bladder Hyperalgesia via TRPA1 Regulation. J. Pain.

[257] Bohlius J., Bohlke K., Lazo-Langner A. (2019). Management of Cancer-Associated Anemia With Erythropoiesis-Stimulating Agents: ASCO/ASH Clinical Practice Guideline Update. J. Oncol. Pract..

[258] Aapro M., Beguin Y., Bokemeyer C., Dicato M., Gascón P., Glaspy J., Hofmann A., Link H., Littlewood T., Ludwig H. (2018). Management of anaemia and iron deficiency in patients with cancer: ESMO Clinical Practice Guidelines. Ann. Oncol..

[259] Katsarou A., Pantopoulos K. (2018). Hepcidin Therapeutics. Pharmaceuticals.

[260] Zhou L., Nguyen A.N., Sohal D., Ying Ma J., Pahanish P., Gundabolu K., Hayman J., Chubak A., Mo Y., Bhagat T.D. (2008). Inhibition of the TGF-beta receptor I kinase promotes hematopoiesis in MDS. Blood.

[261] Verma A., Suragani R.N.V.S., Aluri S., Shah N., Bhagat T.D., Alexander M.J., Komrokji R., Kumar R. (2020). Biological basis for efficacy of activin receptor ligand traps in myelodysplastic syndromes. J. Clin. Invest..

[262] Teixeira A.F., ten Dijke P., Zhu H.-J. (2020). On-Target Anti-TGF-β Therapies Are Not Succeeding in Clinical Cancer Treatments: What Are Remaining Challenges?. Front. Cell Dev. Biol..

[263] Hu J., Liu J., Xue F., Halverson G., Reid M., Guo A., Chen L., Raza A., Galili N., Jaffray J. (2013). Isolation and functional characterization of human erythroblasts at distinct stages: Implications for understanding of normal and disordered erythropoiesis in vivo. Blood.

[264] Elvarsdóttir E.M., Mortera-Blanco T., Dimitriou M., Bouderlique T., Jansson M., Hofman I.J.F., Conte S., Karimi M., Sander B., Douagi I. (2020). A three-dimensional in vitro model of erythropoiesis recapitulates erythroid failure in myelodysplastic syndromes. Leukemia.

[265] Fenaux P., Platzbecker U., Mufti G.J., Garcia-Manero G., Buckstein R., Santini V., Díez-Campelo M., Finelli C., Cazzola M., Ilhan O. (2020). Luspatercept in Patients with Lower-Risk Myelodysplastic Syndromes. N. Engl. J. Med..

[266] Cappellini M.D., Viprakasit V., Taher A.T., Georgiev P., Kuo K.H.M., Coates T., Voskaridou E., Liew H.-K., Pazgal-Kobrowski I., Forni G.L. (2020). A Phase 3 Trial of Luspatercept in Patients with Transfusion-Dependent β-Thalassemia. N. Engl. J. Med..

[267] Cappellini M.D., Porter J., Origa R., Forni G.L., Voskaridou E., Galactéros F., Taher A.T., Arlet J.-B., Ribeil J.-A., Garbowski M. (2019). Sotatercept, a novel transforming growth factor β ligand trap, improves anemia in β-thalassemia: A phase II, open-label, dose-finding study. Haematologica.

[268] Raftopoulos H., Laadem A., Hesketh P.J., Goldschmidt J., Gabrail N., Osborne C., Ali M., Sherman M.L., Wang D., Glaspy J.A. (2016). Sotatercept (ACE-011) for the treatment of chemotherapy-induced anemia in patients with metastatic breast cancer or advanced or metastatic solid tumors treated with platinum-based chemotherapeutic regimens: Results from two phase 2 studies. Support. Care Cancer.

[269] Morrell N.W., Bloch D.B., ten Dijke P., Goumans M.-J.T.H., Hata A., Smith J., Yu P.B., Bloch K.D. (2016). Targeting BMP signalling in cardiovascular disease and anaemia. Nat. Rev. Cardiol..

[270] Steinbicker A.U., Sachidanandan C., Vonner A.J., Yusuf R.Z., Deng D.Y., Lai C.S., Rauwerdink K.M., Winn J.C., Saez B., Cook C.M. (2011). Inhibition of bone morphogenetic protein signaling attenuates anemia associated with inflammation. Blood.

[271] Mayeur C., Kolodziej S.A., Wang A., Xu X., Lee A., Yu P.B., Shen J., Bloch K.D., Bloch D.B. (2015). Oral administration of a bone morphogenetic protein type I receptor inhibitor prevents the development of anemia of inflammation. Haematologica.

[272] Wannamaker W., Davies R., Namchuk M., Pollard J., Ford P., Ku G., Decker C., Charifson P., Weber P., Germann U.A. (2007). (S)-1-((S)-2-{[1 -(4-amino-3-chloro-phenyl)-methanoyl]-amino}-3,3-dimethyl-butanoyl)-pyrrolidine-2-carboxylic acid ((2R,3S)-2-ethoxy-5-oxo-tetrahydro-furan-3-yl)-amide (VX-765), an orally available selective interleukin (IL)-converting enzyme/caspase-1 inhibitor, exhibits potent anti-inflammatory activities by inhibiting the release of IL-1beta and IL-18. J. Pharm. Exp. Ther..

[273] Hu P., Nebreda A.R., Hanenberg H., Kinnebrew G.H., Ivan M., Yoder M.C., Filippi M.-D., Broxmeyer H.E., Kapur R. (2018). P38α/JNK signaling restrains erythropoiesis by suppressing Ezh2-mediated epigenetic silencing of Bim. Nat. Commun..

[274] Martínez-Limón A., Joaquin M., Caballero M., Posas F., de Nadal E. (2020). The p38 Pathway: From Biology to Cancer Therapy. Int. J. Mol. Sci..

[275] Tamura K., Sudo T., Senftleben U., Dadak A.M., Johnson R., Karin M. (2000). Requirement for p38α in Erythropoietin Expression: A Role for Stress Kinases in Erythropoiesis. Cell.

[276] Kuhrt D., Wojchowski D.M. (2015). Emerging EPO and EPO receptor regulators and signal transducers. Blood.

[277] James C., Ugo V., Le Couédic J.-P., Staerk J., Delhommeau F., Lacout C., Garçon L., Raslova H., Berger R., Bennaceur-Griscelli A. (2005). A unique clonal JAK2 mutation leading to constitutive signalling causes polycythaemia vera. Nature.

[278] Libani I.V., Guy E.C., Melchiori L., Schiro R., Ramos P., Breda L., Scholzen T., Chadburn A., Liu Y., Kernbach M. (2008). Decreased differentiation of erythroid cells exacerbates ineffective erythropoiesis in beta-thalassemia. Blood.

[279] Talpaz M., Kiladjian J.-J. (2021). Fedratinib, a newly approved treatment for patients with myeloproliferative neoplasm-associated myelofibrosis. Leukemia.

[280] Hosseini A., Gharibi T., Marofi F., Javadian M., Babaloo Z., Baradaran B. (2020). Janus kinase inhibitors: A therapeutic strategy for cancer and autoimmune diseases. J. Cell. Physiol..

[281] Hua H., Kong Q., Zhang H., Wang J., Luo T., Jiang Y. (2019). Targeting mTOR for cancer therapy. J. Hematol. Oncol..

[282] Zhang X., Campreciós G., Rimmelé P., Liang R., Yalcin S., Mungamuri S.K., Barminko J., D’Escamard V., Baron M.H., Brugnara C. (2014). FOXO3-mTOR metabolic cooperation in the regulation of erythroid cell maturation and homeostasis. Am. J. Hematol..

[283] Franco S.S., De Falco L., Ghaffari S., Brugnara C., Sinclair D.A., Matte A., Iolascon A., Mohandas N., Bertoldi M., An X. (2014). Resveratrol accelerates erythroid maturation by activation of FoxO3 and ameliorates anemia in beta-thalassemic mice. Haematologica.

[284] Sibon D., Coman T., Rossignol J., Lamarque M., Kosmider O., Bayard E., Fouquet G., Rignault R., Topçu S., Bonneau P. (2019). Enhanced Renewal of Erythroid Progenitors in Myelodysplastic Anemia by Peripheral Serotonin. Cell Rep..

[285] Amireault P., Hatia S., Bayard E., Bernex F., Collet C., Callebert J., Launay J.-M., Hermine O., Schneider E., Mallet J. (2011). Ineffective erythropoiesis with reduced red blood cell survival in serotonin-deficient mice. Proc. Natl. Acad. Sci. USA.

[286] Platten M., Nollen E.A.A., Röhrig U.F., Fallarino F., Opitz C.A. (2019). Tryptophan metabolism as a common therapeutic target in cancer, neurodegeneration and beyond. Nat. Rev. Drug Discov..

[287] Stein E.M., DiNardo C.D., Pollyea D.A., Fathi A.T., Roboz G.J., Altman J.K., Stone R.M., DeAngelo D.J., Levine R.L., Flinn I.W. (2017). Enasidenib in mutant IDH2 relapsed or refractory acute myeloid leukemia. Blood.

[288] Dutta R., Zhang T.Y., Köhnke T., Thomas D., Linde M., Gars E., Stafford M., Kaur S., Nakauchi Y., Yin R. (2020). Enasidenib drives human erythroid differentiation independently of isocitrate dehydrogenase 2. J. Clin. Invest..

[289] Stein E.M., DiNardo C.D., Fathi A.T., Pollyea D.A., Stone R.M., Altman J.K., Roboz G.J., Patel M.R., Collins R., Flinn I.W. (2019). Molecular remission and response patterns in patients with mutant-IDH2 acute myeloid leukemia treated with enasidenib. Blood.

[290] Li C., Zhu F., Xu C., Xiao P., Wen J., Zhang X., Wu B. (2019). Dangguibuxue decoction abolishes abnormal accumulation of erythroid progenitor cells induced by melanoma. J. Ethnopharmacol..

[291] Gonzalez-Menendez P., Romano M., Yan H., Deshmukh R., Papoin J., Oburoglu L., Daumur M., Dumé A.S., Phadke I., Mongellaz C. (2021). An IDH1-vitamin C crosstalk drives human erythroid development by inhibiting pro-oxidant mitochondrial metabolism. Cell Rep..

[292] Steenbrugge J., De Jaeghere E.A., Meyer E., Denys H., De Wever O. (2021). Splenic Hematopoietic and Stromal Cells in Cancer Progression. Cancer Res..

[293] Levy L., Mishalian I., Bayuch R., Zolotarov L., Michaeli J., Fridlender Z.G. (2015). Splenectomy inhibits non-small cell lung cancer growth by modulating anti-tumor adaptive and innate immune response. OncoImmunology.

[294] Sano T., Sasako M., Mizusawa J., Yamamoto S., Katai H., Yoshikawa T., Nashimoto A., Ito S., Kaji M., Imamura H. (2017). Randomized Controlled Trial to Evaluate Splenectomy in Total Gastrectomy for Proximal Gastric Carcinoma. Ann. Surg..

[295] Fallah J., Olszewski A.J. (2019). Diagnostic and therapeutic splenectomy for splenic lymphomas: Analysis of the National Cancer Data Base. Hematology.

[296] Yu W., Choi G.S., Chung H.Y. (2006). Randomized clinical trial of splenectomy versus splenic preservation in patients with proximal gastric cancer. Br. J. Surg..

[297] Crawford J., Cella D., Cleeland C.S., Cremieux P.Y., Demetri G.D., Sarokhan B.J., Slavin M.B., Glaspy J.A. (2002). Relationship between changes in hemoglobin level and quality of life during chemotherapy in anemic cancer patients receiving epoetin alfa therapy. Cancer.

[298] Caro J.J., Salas M., Ward A., Goss G. (2001). Anemia as an independent prognostic factor for survival in patients with cancer: A systemic, quantitative review. Cancer.

[299] Ludwig H., Van Belle S., Barrett-Lee P., Birgegård G., Bokemeyer C., Gascón P., Kosmidis P., Krzakowski M., Nortier J., Olmi P. (2004). The European Cancer Anaemia Survey (ECAS): A large, multinational, prospective survey defining the prevalence, incidence, and treatment of anaemia in cancer patients. Eur. J. Cancer.

[300] Petrova V., Annicchiarico-Petruzzelli M., Melino G., Amelio I. (2018). The hypoxic tumour microenvironment. Oncogenesis.

[301] Haemmerle M., Stone R.L., Menter D.G., Afshar-Kharghan V., Sood A.K. (2018). The Platelet Lifeline to Cancer: Challenges and Opportunities. Cancer Cell.

[302] Lucotti S., Muschel R.J. (2020). Platelets and Metastasis: New Implications of an Old Interplay. Front. Oncol..

[303] Fan J., Slowikowski K., Zhang F. (2020). Single-cell transcriptomics in cancer: Computational challenges and opportunities. Exp. Mol. Med..

[304] Slyper M., Porter C.B.M., Ashenberg O., Waldman J., Drokhlyansky E., Wakiro I., Smillie C., Smith-Rosario G., Wu J., Dionne D. (2020). A single-cell and single-nucleus RNA-Seq toolbox for fresh and frozen human tumors. Nat. Med..

[305] Wynn J.L., Scumpia P.O., Stocks B.T., Romano-Keeler J., Alrifai M.W., Liu J.-H., Kim A.S., Alford C.E., Matta P., Weitkamp J.-H. (2015). Neonatal CD71^+^ Erythroid Cells Do Not Modify Murine Sepsis Mortality. J. Immunol..

[306] Sennikov S.V., Injelevskaya T.V., Krysov S.V., Silkov A.N., Kovinev I.B., Dyachkova N.J., Zenkov A.N., Loseva M.I., Kozlov V.A. (2004). Production of hemo- and immunoregulatory cytokines by erythroblast antigen^+^ and glycophorin A^+^ cells from human bone marrow. BMC Cell Biol..

